# Two Evolutionary Histories in the Genome of Rice: the Roles of Domestication Genes

**DOI:** 10.1371/journal.pgen.1002100

**Published:** 2011-06-09

**Authors:** Ziwen He, Weiwei Zhai, Haijun Wen, Tian Tang, Yu Wang, Xuemei Lu, Anthony J. Greenberg, Richard R. Hudson, Chung-I Wu, Suhua Shi

**Affiliations:** 1 State Key Laboratory of Biocontrol and Guangdong Key Laboratory of Plant Resources, Sun Yat-Sen University, Guangzhou, China; 2 Laboratory of Disease Genomics and Individualized Medicine, Beijing Institute of Genomics, Chinese Academy of Sciences, Beijing, China; 3 Graduate University of the Chinese Academy of Sciences, Beijing, China; 4 Department of Molecular Biology and Genetics, Cornell University, Ithaca, New York, United States of America; 5 Department of Ecology and Evolution, University of Chicago, Chicago, Illinois, United States of America; 6 CAS Key Laboratory of Genome Sciences and Information, Beijing Institute of Genomics, Chinese Academy of Sciences, Beijing, China; University of Georgia, United States of America

## Abstract

Genealogical patterns in different genomic regions may be different due to the joint influence of gene flow and selection. The existence of two subspecies of cultivated rice provides a unique opportunity for analyzing these effects during domestication. We chose 66 accessions from the three rice taxa (about 22 each from *Oryza sativa indica*, *O. sativa japonica*, and *O. rufipogon*) for whole-genome sequencing. In the search for the signature of selection, we focus on low diversity regions (LDRs) shared by both cultivars. We found that the genealogical histories of these overlapping LDRs are distinct from the genomic background. While *indica* and *japonica* genomes generally appear to be of independent origin, many overlapping LDRs may have originated only once, as a result of selection and subsequent introgression. Interestingly, many such LDRs contain only one candidate gene of rice domestication, and several known domestication genes have indeed been “rediscovered” by this approach. In summary, we identified 13 additional candidate genes of domestication.

## Author Summary

The origin of two cultivated rice *Oryza sativa indica* and *O. sativa japonica* has been an interesting topic in evolutionary biology. Through whole-genome sequencing, we show that the rice genome embodies two different evolutionary trajectories. Overall genome-wide pattern supports a history of independent origin of two cultivars from their wild population. However, genomic segments bearing important agronomic traits originated only once in one population and spread across all cultivars through introgression and human selection. Population genetic analysis allows us to pinpoint 13 additional candidate domestication genes.

## Introduction

A main objective in the study of natural and domesticated species is to systematically identify genomic regions that have been influenced by selection. A strategy that is effective but not commonly used is to search for genomic regions with an unusual genealogical history [1], [2]. During speciation or domestication, if gene flow continues between diverging populations, selection may play a large role in shaping the genealogies of different parts of the same genome. For example, mutations that contribute to local adaptation may spread in some populations but not others, leading to a higher level of differentiation at and near the genes for local adaptation [3]–[5]. In contrast, mutations that are universally selected may spread among populations more rapidly than neutral variants resulting in reduced differentiation.

The joint action of gene flow and selection could be even stronger in domesticated species than in natural populations as breeders might cross varieties between subspecies that do not readily interbreed in nature. Furthermore, human selection for desired traits is often intense. In this context, Asian cultivated rice (*Oryza sativa*) is of particular value as there are two subspecies, *indica* and *japonica*, which are partially reproductively isolated [6]. The origin of cultivated rice is therefore a question of how human selection created the two types of rice [7]. Because phylogenetic studies tend to support the independent domestication hypothesis [8]–[11], we may have the unusual opportunity to analyze the course of evolution twice from the same common ancestor, the Asian wild rice *O. rufipogon* [6].

If *indica* and *japonica* were independently domesticated, then a genome-wide pattern is expected. However, some loci in rice show patterns of variation inconsistent with the independent domestication hypothesis. For example, the *sh4* locus which is responsible for the reduction in grain shattering among cultivars is fixed in both subspecies for the same allele [12]–[14]. The genealogy suggests a single domestication event with respect to the *sh4* locus and the new allele subsequently spread to all cultivars. In this study, we take a whole-genome approach to sequencing 66 accessions of rice in order to answer these questions: i) which genomic regions in rice exhibit a genealogy distinct from the rest of the genome? ii) how do these regions reflect the process of domestication under artificial selection? and iii) how many domestication genes can be identified in these regions?

## Results

In this study, we first surveyed genome wide diversity pattern by sequencing multiple lines of *O. rufipogon*, *O. sativa indica* and *O. sativa japonica*. While second generation technologies, such as Illumina-Solexa-GA and ABI-SOLiD make the task feasible, they are more error-prone than the conventional Sanger method [15], [16]. Therefore, to distinguish true polymorphisms from sequencing errors, we used both platforms for sequencing and retained only the polymorphic sites identified by both methods and discarded singletons, a procedure that is quite effective at significantly driving down false positives [17] (see Materials and Methods and Text S1).

We sequenced pooled DNA samples of each subspecies (21–23 accessions per subspecies used, Table S1) with the coverage about 30X for each sample, or 1.5X per accession (Table S2). Although it may seem more informative to sequence each accession individually, the gain in information, for example about linkage disequilibrium, is achieved only when the coverage is deep for each line [18]. In fact, if the objective is to estimate genetic diversity in the population, data from mixed samples can often be as informative as data from individual lines [18].

We first estimate genetic diversity (θ) genome-wide using a method we describe in detail in another paper (He et al, in submission). We use Watterson's estimator of θ [19], which is based on the number of sites that are polymorphic. In Table 1, S is the number of such segregating sites in a given region, while S_>1_ is the number of sites excluding singletons; S_>2_ estimates further exclude doubletons. The estimates from the combined data are lower than those based on either SOLiD or GA data alone and are close to previous estimates based on conventional sequencing of selected genes [20], [21]. Overall, *indica* retains much more genetic diversity than *japonica*, as has been reported in the literature[22]. For the rest of this study, we use θ estimates based on the combined GA/SOLiD data with S_>1_. A detailed comparison of various procedures of θ estimation can be found in Table S3.

**Table 1:** Estimated θ per kb for *O. rufipogon*, *indica*, and *japonica* under different schemes of site selection.

Platform	Sites used	*japonica*	*indica*	*O. rufipogon*
GA	S (All sites)	8.55	10.13	11.53
	S_>2_	1.13	4.70	5.30
SOLiD	S (All sites)	13.89	13.98	12.46
	S_>2_	1.64	4.24	4.64
Combined	S_>1_	0.90	3.72	4.04

Only sites whose coverage in GA and SOLiD platform are both 6X or more are used. S is the number of segregating sites in a given region and S>1 counts the same sites but excludes singletons. S>2 excludes doubletons in addition. For the “combined” (GA plus SOLiD) data, S>1 represents keeping sites whose variant appear more than once in both GA and SOLiD data. For a comparison, estimates based on all polymorphic sites are also given (“All sites”). These estimates are greatly inflated due to the excesses in singletons and doubletons, many of which are sequencing errors (See Table S3 for more information).

**Figure 1:**
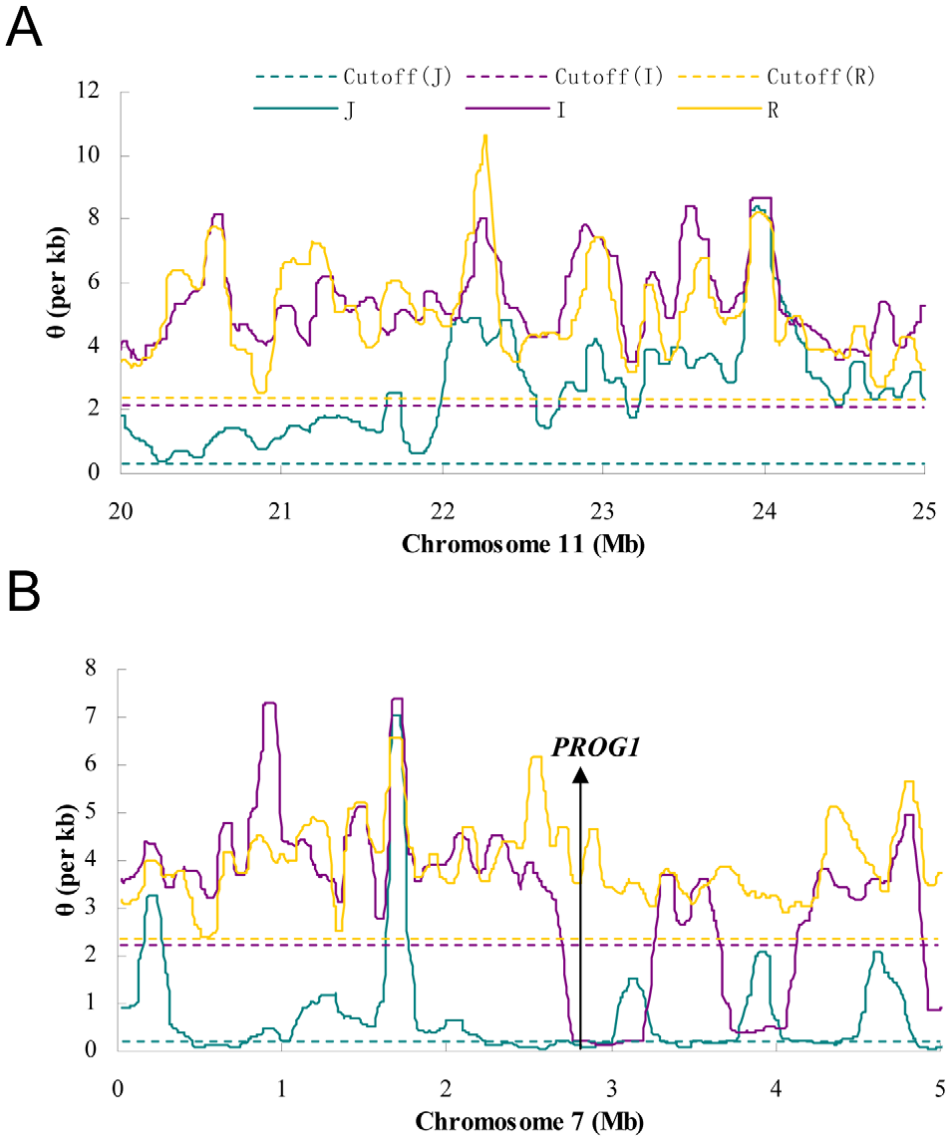
The sliding window profiles of θ in two 5 Mb regions. The window size is 100 kb and step size is 10 kb. The horizontal lines are the cutoffs determined for each subspecies by whole-genome random shuffling. A) A typical region on chromosome 11 where no sub-region is lower than the cutoff in all species. B) A region on chromosome 7 that contains *PROG1*, a locus known to be associated with domestication [24]. Both the *indica* and *japonica* genomes are below the cutoff in the neighborhood (300 kb and 780 kb, respectively) of *PROG1*.

Figure 1 shows diversity estimates from a sliding-window analyses across each genome, with 100 kb windows and steps of 10 kb (See Materials and Methods for details; Window size was chosen based on typical levels of linkage disequilibrium in these species) [23]. Figure 1 gives two example profiles of θ, 5 Mb each. Panel (A) is a region with normal diversity. Genome wide low diversity cutoffs are plotted as the dashed lines for three species respectively. For each genome, in order to explore the heterogeneity in local variation, we chose a cutoff to identify regions of low diversity based on the characteristics of each genome. While there are many potential ways to select a cutoff value, a simple one determined by shuffling 1 kb segments of the entire genome will be used in our analysis. By this method, the lowest value among all windows was chosen as the cutoff (see Materials and Methods). Selection, demography and selfing may all generate genomic regions of unexpectedly low genetic diversity. We used other means of selecting the cutoffs and, as shown in Text S1 (section F), the main conclusions remain the same. Panel (B) shows the position of *PROG1* which controls a key transition from prostrate to erect growth during domestication [24]. The *PROG1* locus falls into a region of low polymorphism in both *indica* and *japonica*. A plot for the entire genome is given in Figure S1.

### Low diversity regions (LDRs) in domesticated rice

Table 2 summarises the number of genomic regions with lower diversity than genome wide cutoff values for each of the three taxa. The number of such low diversity regions (LDRs) in *O. rufipogon* decreases quickly if we increase window size. Only four LDRs in *O. rufipogon* are larger than 200 kb, accounting for 0.25% of the genome. In contrast, 6.15% of the *indica* genome falls in LDRs larger than 200 kb and more than 25% of the *japonica* genome appears to have too little polymorphism. Large genomic segments devoid of genetic diversity are observed in multiple domesticated animals [25]. The excess of LDRs in the cultivated rice is presumably attributable to domestication, which includes artificial selection, population size reduction, introgression and selfing.

**Table 2:** Numbers of contigs in different size categories where θ is lower than the cutoff.

Contig size (kb)	Number of contigs (% genome)			
	*japonica* (J)	*indica* (I)	overlapping regions (I and J)	*O. rufipogon*
<100	64	60	40	77
100∼200	96	44	27	31
200∼300	49	28	11	4
300∼400	22	17	6	0
400∼500	33	5	2	0
500∼600	9	2	2	0
≥600	53	7	2	0
<200 kb	160 (4.90%)	104 (2.75%)	67 (1.60%)	108 (2.45%)
≥200 kb	166 (26.38%)	59 (6.15%)	23 (2.35%)	4 (0.25%)

Common regions are windows overlapping between *indica* and *japonica*. The cutoff is determined for each subspecies by whole-genome random shuffling of 1 kb segments (see Materials and Methods). The cutoff values (θ per kb) are 0.215 for *japonica*, 2.153 for *indica* and 2.343 for *O. rufipogon*.

While it is tempting to associate LDRs with selective sweeps under artificial selection, other forces of domestication must be considered as well. In particular, since both cultivars are self-pollinators whereas *O. rufipogon* is largely an outcrossing species [6], [26], population bottlenecks together with selfing are likely to generate genomic segments with reduced polymorphism. To assess whether these forces are sufficient to explain the excess of LDRs in the domesticated cultivars, we performed a series of simulations (see Text S1 section B for details). These simulations indeed indicate that for plausible levels of population-size reduction and effect of selfing on recombination, it is possible to observe the patterns of genomic diversity we see in the data.

Since demography and selfing are confounding factors, inference of selective sweeps cannot be justified solely by the prevalence of low diversity regions. If selection has affected the genomes of cultivated rice, this will have to be determined from the patterns of genetic variation within LDRs.

To tease apart the evolutionary forces that influence LDRs, we took advantage of the existence of two subspecies of domesticated rice. Since both domesticated sub-species were selected for a similar suite of characteristics, it was reasonable to hypothesize that the same genes might be affected. We therefore identified LDRs that are spatially overlapping between *indica* and *japonica* (referred to as “overlapping LDRs”). Overlapping LDRs could happen by chance, by independent but convergent selection in the two subspecies, or by introgression from one subspecies to the other. The genealogical patterns of these overlapping LDRs, in comparison with the genomic background, should be informative.

For convenience, we will use R for *O. rufipogon*, I for *O. sativa indica* and J for *O. sativa japonica* to indicate the genomic background, and R*, I*, J* to indicate overlapping LDRs. To explore potentially different genealogical histories between different parts of the genome, we first used a simplest method by calculating genetic distances for overlapping LDRs and for whole-genome sequences, respectively. The genetic distance is the average distance between two sequences, each randomly chosen from the populations of interest (see Materials and Methods), and is a simple and well characterised method for assessing relationships among populations.

**Figure 2:**
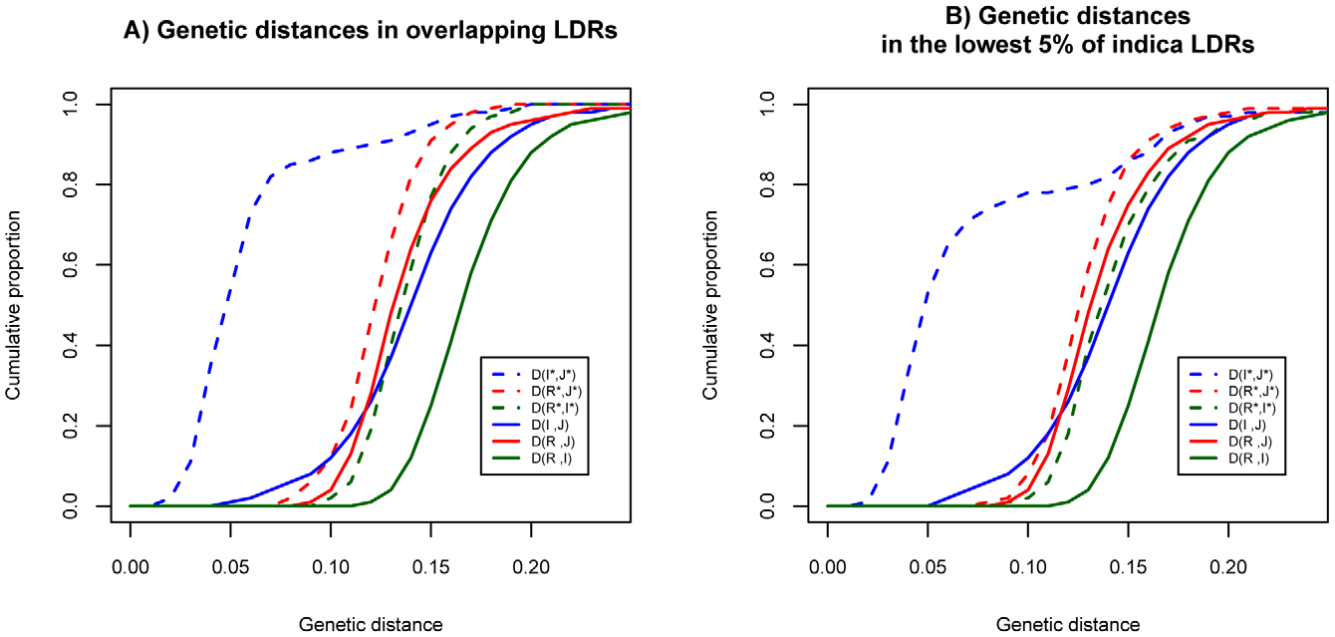
Distributions of genetic distances between populations in the genomic background and LDRs. A) The cumulative distributions at overlapping LDRs (dashed curves) and genome background (solid curves). B) The cumulative distributions at bottom 5% of LDRs in *indica* (dashed curves) and genome background (solid curves). We use R for *O. rufipogon*, I for *indica* and J for *japonica* to indicate the genomic background. Overlapping LDRs (in panel A) or bottom 5% of LDRs (in panel B) in these species are designated by I*, J* and R* respectively.

Figure 2 displays the cumulative distributions for the distances. For the genomic background, the genetic distances are very similar in the three pair-wise comparisons (solid lines). In light of the independent history of the two cultivars generally accepted in the literature, similar distances between wild species and cultivars are expected. The genetic distance between R and I is slightly larger than those of the other two comparisons because these two subspecies are the more polymorphic ones. (Hence, the coalescence time of some alleles from R and I could be older than the divergence time of the two subspecies.)

**Figure 3:**
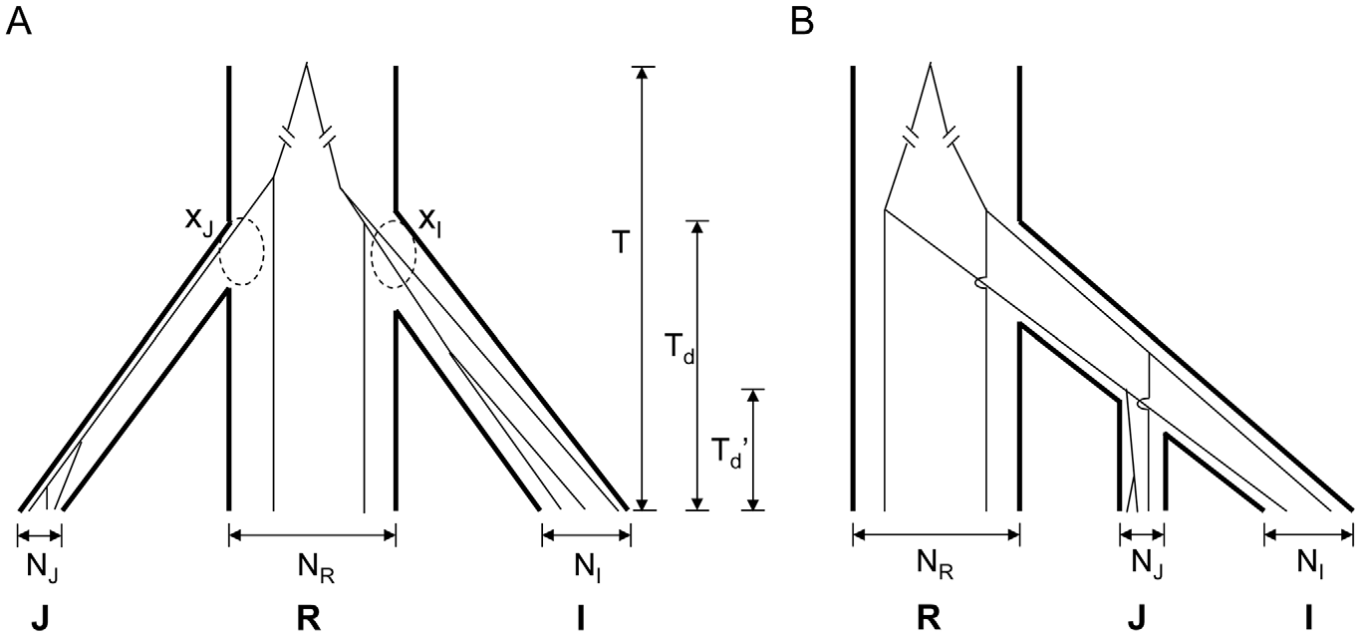
Two models for the domestication of *indica* (I) and *japonica* (J). A) Independent domestication – In the simplest form of independent domestication, *indica* and *japonica* were separately domesticated from *O. rufipogon* at about the same time, resulting in a trifurcation phylogeny. The most recent common ancestor of three taxa was time T from present. The two dashed circles highlight the coalesced lineages (x_I_ and x_J_, respectively) at the time of domestication, T_d_. Branch widths reflect the relative population sizes (N_I_, N_R_ and N_J_) of the three taxa. B) Sequential domestication – In this model, *indica* and *japonica* share a common history of domestication (T_d_'), and they are most closely related to each other.

Interestingly, in the LDRs, I and J are genetically closer to each other than each is to *O. rufipogon* (Figure 2A, dashed lines). Moreover, this observation that I and J are unusually closely related appears to be a general property of regions of reduced genetic diversity. For example, the lowest 5% LDRs chosen from *indica* alone, exhibit very similar patterns as the overlapping LDRs (see Figure 2B). The divergence patterns in Figure 2 suggest different evolutionary histories between genomic background and overlapping LDRs. More specifically, divergence in the genomic background among the three subspecies appears to be commensurate with the widely-held view of independent domestication of I and J from O. *rufipogon* (Figure 3A). However, the closer relationship between the two cultivars in overlapping LDRs hints support for sequential domestication (Figure 3B). These hints are examined closely below.

### Different evolutionary histories in the same genome—observations versus simulations

The analysis above did not incorporate within-subspecies polymorphism. To take into account polymorphisms in the analysis, we used the Fst statistic [27]. Fst reflects the proportion of total genetic diversity that is due to among-population differentiation. Polymorphic sites with Fst  = 0 exhibit no differentiation, while those with Fst  = 1 show complete differentiation with no common alleles among populations. Since mosaic genealogies can be statistically complex, we determined the statistical confidence by comparing the observation with extensive coalescence simulations, which use information on standing polymorphisms.

**Figure 4:**
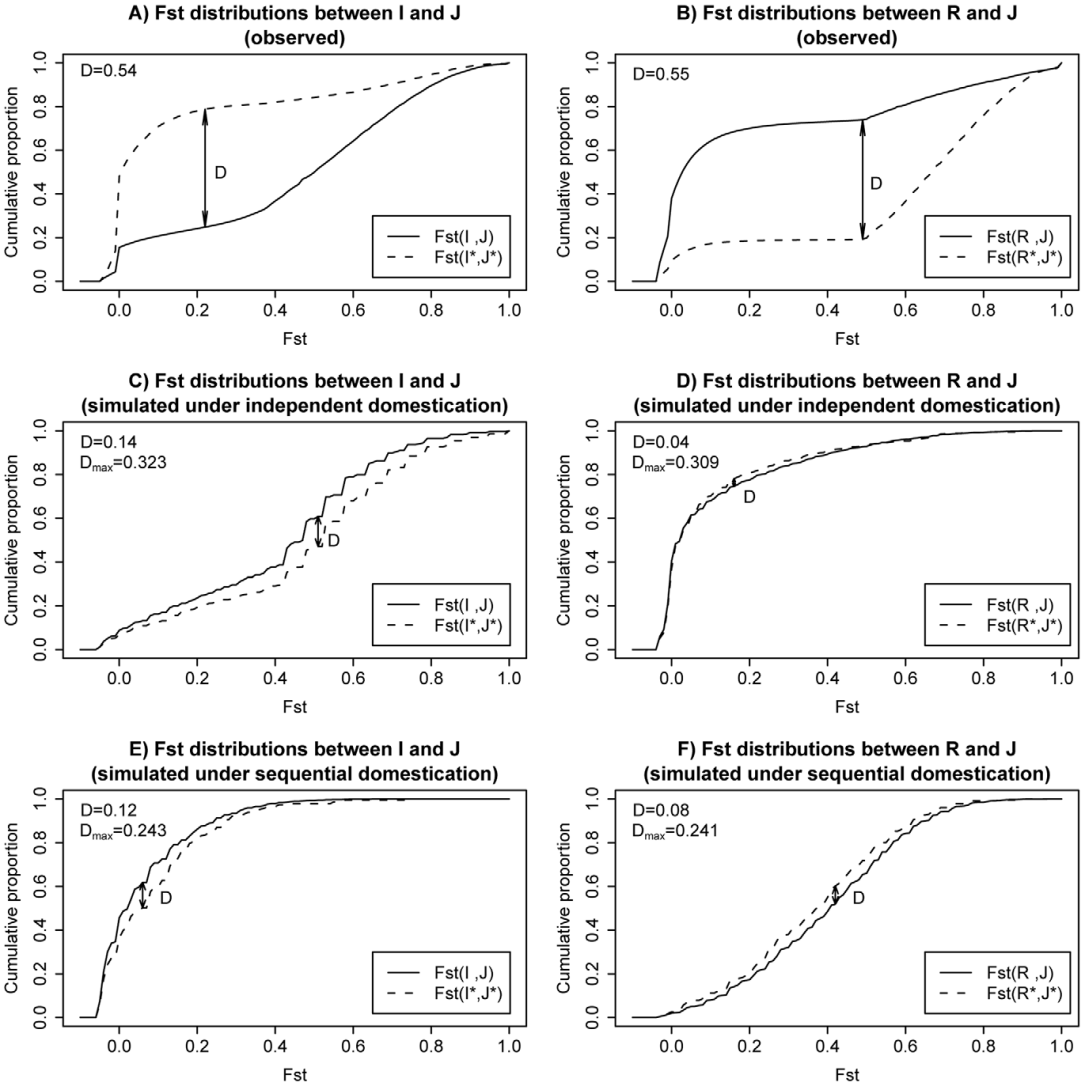
Cumulative plots for Fst distributions in observed data and two example simulations. A) Observed cumulative plot for Fst between I and J; Fst distribution for overlapping LDRs are plotted in dashed lines. Solid lines are used for genome background. B) Observed cumulative plot for Fst between R and J. C) Simulated cumulative plot for Fst between I and J under an independent domestication history. D) Simulated cumulative plot for Fst between R and J under an independent domestication history. E) Simulated cumulative plot for Fst between I and J under a sequential domestication history. F) Simulated cumulative plot for Fst between R and J under a sequential domestication history. D measures the maximal distances between the two plotted cumulative distributions in each panel (see main text). Both observed D value in real data and maximal value across simulated replicates are shown in left upper corner of each panel.

The observed cumulative distributions of Fst are shown in Figure 4A (for I vs. J) and Figure 4B (for R vs. J). In each panel, the distributions of the whole genome and overlapping LDRs are represented by the solid and dotted line, respectively. Similar to what we observe in Figure 2, overlapping LDRs show a pattern of population differentiation distinct from that of the genome background. We measured the largest distance between the dotted curve (for overlapping LDRs) and the solid curve (genomic background), marked D in Figure 4A and 4B. The observed D value is given in the upper left corner of each panel.

To find out whether the observed D's in Figure 4A and 4B are compatible with neutral demographical models, we performed coalescent simulations. The simulations were done under either the independent domestication model of Figure 3A or the sequential domestication model of Figure 3B. We explored a wide range of parameter combinations. The simulation scheme and parameters chosen are described in detail in Text S1 (section D). Representative results are shown in Figure. 4C–4F.

As shown in Figure 4C–4F, the dotted and solid curves are not very different under one single evolutionary history, regardless of the particular model of demography. The simulated D's are much smaller than those observed in Figure 4A and 4B. For a statistical test of D_Fst_, we simulated 4000 replicates from a set of 8 parameter combinations (see Text S1 section D). The maximal D_Fst_ from the 4000 simulations is given in each panel as well. In all cases, the maximal D_Fst_ is far smaller than the observed value. Therefore, the genealogy of overlapping LDRs as observed in Figure 4A and 4B is not likely to result from the same evolutionary history as that of the rest of the genome (Text S1 section D) and is robust to possible ancestral structure in *rufipogon* population (Text S1 section G).

What then might account for the different evolutionary histories in the same genome? The solid curves in the observation (Figure 4A and 4B) appear to agree with the simulations under the independent domestication model of Figure 4C and 4D. In contrast, the dotted curves for the observations seem to follow the sequential domestication model of Figure 4E and 4F. In short, while the genomic background follows the independent domestication model, consistent with the accepted view of rice domestication, the genealogy of overlapping LDRs follows the sequential domestication model.

There may be two explanations for the observed closer relationship between two cultivars in overlapping LDRs. In the first explanation, independent selection for the same trait drove the same set of alleles in *rufipogon* to high frequency in the domesticated species. However, since linkage disequilibrium in the wild species is limited, typically spanning only a few kilobases [23] and is much less than the length of overlapping LDRs, selection for the same focal allele is not likely to drag the same set of nearby variants to fixation in the two subspecies. A second, and perhaps more likely, explanation is that genomic segments were selected in one subspecies and subsequently introgressed into the other. It seems plausible that breeders through the ages hybridized varieties in order to introduce desired traits from one variety to others [28].

We should note that the observed and simulated results of Figure 4 is based on sites where Fst (R, I) ≥0.5. At sites where R and I (the two more highly polymorphic taxa) are not strongly differentiated, there is little statistical resolution in genealogies between models of Figure 3A and 3B. At those sites, the difference in genealogies between LDRs and the genomic background cannot be easily observed. Hence, we focused on sites that are sufficient differentiated between R and I with Fst (R, I) ≥0.5 and asked if J is significantly more closely related to I (Figure 3B) or nearly equally related to R and I (Figure 3A). The conclusions are the same when all sites are used (see Figure S2), but the resolution is lower, as expected. We also note that a separate analysis that switches I and J yields the same conclusion as Figure 4. That analysis asks whether I is closer to J or R at sites where Fst (R, J) ≥0.5. We prefer the analysis presented in Figure 4 because I and R are comparably polymorphic and much more so than J. This property makes it easier to see the predicted outcome in Figure 4 under either model of Figure 3A or Figure 3B.

### Genomic regions enriched for genes of domestication

If the hypothesis of frequent introgressions between *indica* and *japonica* [28], [29] is correct, then overlapping LDRs may have played an important role in rice domestication. These overlapping LDRs may be enriched for genes underlying interesting traits in both *indica* and *japonica*. Therefore, we focused on the 61 genomic regions where Fst(I*, J*)'s are significantly smaller than Fst (R*, I*)'s and Fst (R*, J*)'s at the 5% nominal level by the Kolmogorov-Smirnov test [30] (Table S4). These 61 genomic segment account for about 3% of the rice genome and 86.7% of all the overlapping LDRs (Table 2 and Table S4).

**Figure 5:**
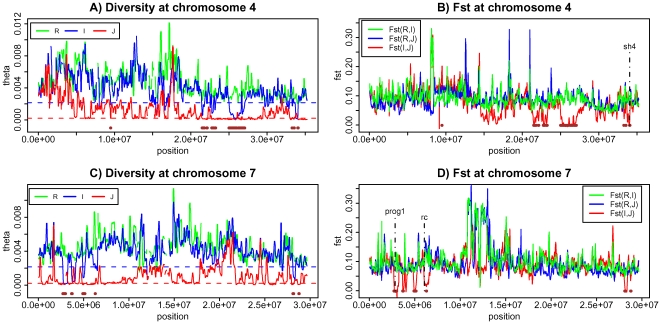
Genetic diversity and population differentiation at chromosome 4 and 7. A) Genetic diversity at chromosome 4 for three species. B) Population differentiation at chromosome 4 for all three pair-wise comparisons C) Genetic diversity at chromosome 7 for three species. D) Population differentiation at chromosome 7 for all three pair-wise comparisons. Brown horizontal bars are the overlapping low diversity regions identified in this study.

For a positive control, genes that are known to delineate domesticated rice from their wild progenitors by an important trait should fall in these regions (Figure 5 and Table S4). The *sh4* gene, responsible for seed shattering [12]–[14], and *PROG1*, associated with the transition from the prostrate growth in the wild rice to the erect growth of cultivars [24], are the two best examples (Figure 5). Both are indeed in one of the 61 regions (Table S4). A third gene (*Rc*) responsible for the white grain pericarp in cultivars [28], [31] is another possibility although the association between the phenotype and the cultivars is incomplete. *Rc* is also close to one of the 61 overlapping LDRs identified (Figure 5 and Text S1 section E).

We wished to identify, from this analysis of LDRs, new candidate genes of rice domestication. We chose candidate genes within the 61 regions that have at least one nonsynonymous mutation distinguishing (I, J) from R. Specifically, we required both Fst (I, R) and Fst (J, R) to be >0.8 but Fst (I, J) <0.1 at these sites. It should be noted that such a gene is not expected in every of the 61 regions since adjacent regions may not have been independently derived. For example, a large portion of a chromosome could have been introgressed initially when a single gene of domestication spread among cultivars. This large region was then broken into many smaller LDRs by recombination (Figure S1). In that case, several LDRs may have resulted from one single introgression.

Among the 61 regions, 20 regions contain at least one gene fulfilling the Fst criteria. Interestingly, 13 of these 20 regions are represented by a single candidate gene. Table S5 presents these 13 genes with their putative functions listed; two are of special interest. LOC_Os01g36640 is a candidate gene of disease resistance. Its expression level increases sharply after treatment with *Magnaporthe grisea* suggesting its functional role in blast fungus resistance [32]. Similar to a previously cloned gene Pi-ta, this gene also has one single amino acid difference between the resistant and susceptible alleles [33]. LOC_Os03g44710 encodes a YABBY domain-containing protein. In Arabidopsis, members of the YABBY gene family specify abaxial cell fate. Thus LOC_Os03g44710 may contribute to the architectural difference between wild and cultivated rice [34].

In all, we have identified 13 genes that bear the population genetic signature of having been selected in one domesticated subspecies and introgressed to the other subsequently. Each of these genes is embedded in an overlapping LDR between the two subspecies. To ensure that the inference of these 13 candidate gene regions was not biased by the relatively small sample size of roughly 22 accessions in each subspecies, we examined a much larger collection of accessions published recently [35]. This collection consists of 373 *indica* and 131 *japonica* lines, each of which lightly sequenced (about 1 X coverage). In this large dataset, the average diversity of these 13 regions in *indica* is 0.00074 in a genomic background of 0.0016. In *japonica*, the corresponding values are 0.0001 and 0.0006, respectively. Therefore, these 13 candidate regions are indeed much lower in genetic diversity than the genomic background across a very large number of accessions. We should note that, in the larger collection, one of the 13 regions in *indica* shows a relatively high diversity that is twice higher than the average of the rest. This outlier region is marked out in Table S5. Several of these genes are currently being tested for their functional role in delineating cultivars from their wild progenitors.

## Discussion

In this study, we surveyed whole-genome DNA polymorphisms in rice. It is commonly accepted that LDRs are a possible signature of selective sweep and LDRs are indeed more common in the cultivars than in the wild rice in our study. However, because of population bottleneck and selfing, the prevalence of LDRs in the cultivars is also compatible with many purely demographic scenarios.

To address the issue of selection versus demography, we took advantage of the independent domestication of *indica* and *japonica*. We showed, by two different approaches, that some LDRs have an evolutionary history distinct from the rest of the genome. These LDRs, overlapping between the two subspecies and accounting for about 3% of the genome, bear the signature of introgression from one subspecies to the other (Table 2 and Table S4). Such introgressions imply human selection and become the target regions in the search for genes of rice domestication.

Because this analysis aimed at identifying genetic changes that distinguish cultivars, be they landraces or elite accessions in *indica* or *japonica*, from O. *rufipogon*. it would have missed variations that delineate different groups of cultivars, such as Phr-1 [36]. We suspect that the changes identified here may tend to be associated with earlier events in domestication. In general, these genes may be difficult to identify by the conventional means of mapping and cloning. To do that, it would be necessary to show that the traits differentiate most *O. rufipogon* lines from *indica* and *japonica* lines. This requirement would entail laborious and extensive genetic mapping. Hence, a pre-screen for candidate domestication genes by the population genetic analyses shown here could be worthwhile.

The criteria used to construct the list of overlapping LDRs yield both *sh4* and *PROG1* (Table S4), the two best known genes that distinguish wild rice from the cultivars. This predicted gene list (Table S5) should therefore be enriched for domestication genes. As the number of candidate genes associated with each overlapping LDR is often small (one single candidate in many cases), direct testing by transgenic means is well justified.

The main point of this study is that certain LDRs appear to be introgressions driven by positive selection. An interesting, but secondary point, concerns the direction of introgression, i.e., from *japonica* to *indica* or vice versa [29]. While the two types of introgressions may leave different footprints in the polymorphism patterns, the statistical resolution is too weak to be conclusive (Text S1, Section H). Further studies of the haplotype structure near the focal sites may provide an answer to this question [e.g. 29].

## Materials and Methods

### Sample preparation and sequencing

We used 43 lines of *Oryza sativa* including 21 *japonica* and 22 *indica* accessions and 23 lines of *O. rufipogon* in this study (Table S1). Total DNA was extracted from leaves using the CTAB method [37]. For each taxon (*japonica*, *indica*, and *O. rufipogon*), we pooled equal amount of total DNA from all individuals of that taxon for sequencing. Pooled samples were processed with the Illumina Genome Analyser at the Beijing Genomics Institute (Shenzhen), following the manufacturers' instructions. We sequenced each sample using a full run and generated paired-ends reads. We also sequenced the same samples using the ABI SOLiD sequencing platform at Beijing Institute of Genomics (Beijing) (two slides per sample) and generated single-end reads.

### Mapping of sequencing data

Short reads generated by the two platforms were mapped to the reference genome (MSU Rice Genome Annotation Project Release 6.0, http://rice.plantbiology.msu.edu/) using *MAQ* [38]. Only uniquely mapped reads were used for subsequent analysis. The main parameters (-n 2 -a 400 -m 0.002(J)/0.01(I,R) -C 20 -e 200 -N) were used in mapping and parameters (-m 3 -q 20) were used to filter low quality reads in GA data. For SOLiD data, we used parameters (-n 3 -c -m 0.005(J)/0.01(I, R) -C 20 -e 200 -N) in color spaces mapping and parameters (-m 5 -Q 1000 -q 20) to filter low quality reads. To reduce the error rate caused by the low quality sites in reads, we discarded bases where quality values were lower than 15.

### Method of estimating θ

To accurately estimate θ, we had to filter out sequencing errors. We accomplished this by using only variant sites detected by both sequencing platforms and estimating Watterson's θ [19], which does not require knowing allele frequencies (E(S)  =  a_n_θ, where S is the number of segregating sites, a_n_  =  (1+1/2+1/3+….+1/[n-1]) and n is the sample size (n = 21, 22, and 46 in *japonica*, *indica* and *O. rufipogon*, respectively). Many singletons and doubletons are caused by sequencing errors. To minimize the confounding effects of these errors, we used S_>1_ (segregating sites excluding singletons) and S_>2_ (excluding doubletons in addition) to estimate θ. We describe the method in detail in another paper (He et al, in submission).

### Identification of LDRs (low diversity regions)

θ was estimated from the combined GA/SOLiD data across the whole-genome using a sliding window approach. The window size was 100 kb and step size was 10 kb. To identify windows with unusually long stretches of low polymorphism, we calculated cutoff θ values for each of the three taxa separately. We broke the genomes into 1 kb units and randomly shuffled these pieces 200 times, rendering the diversity at each adjacent segment independently. For each shuffled genome, we calculated θ in each 100 kb window and recorded the lowest θ (θ_min_). Among the 200 θ_min_, we selected 10th smallest as the cutoff (hence, *P* = 0.05). The cutoff is defined as the level at which 95% of the simulations do not yield a single 100 kb segment with a θ value below it. Note that in the 5% of the cases where simulations yielded some 100 kb segments below the cutoff the number of such segments is never greater than 2.

### Sliding-window calculations of θ

We set the window size at 100 kb, in keeping with average levels of linkage disequilibrium in the cultivars, or larger when specified. We then let the windows slide along each chromosome by 10 kb steps. We used the S_>1_ of combined data to calculate θ of every window which has 10,000 sites covered at least four reads from both platforms. Most of the 10 kb region is covered by 10 windows and some are not. We thus only retained regions covered by four or more windows, and chose the median θ of these windows to represent each region. If its median θ value was lower than the cutoff, we treated it as a low polymorphism region.

### Genetic distances

For a polymorphic SNP position, allele frequencies in population one are p1 and q1. In population two, the corresponding frequencies are p2 and q2 respectively. Then genetic distance between two populations at this position is p1*q2 +p2*q1. The distance for a genomic segment is the average distance across all SNP positions within this region. This genetic distance measures the average distances for all pairwise comparisons between two sequences each taken at random from two populations. It has range between 0 and 1.

### Calculating Fst

We used the method described by Weir [27] to estimate Fst. For each taxon, we combined the reads from both platforms (Table S2). For a more accurate estimation, we used only high quality bases covered by at least 10 reads in all three taxa (see Mapping of sequencing data). We discarded all sites that had a single mutation in the combined three-species data set.

### Coalescent simulations under different demographic histories

We take two different approaches to the simulations of sequence evolution under either model of rice domestication (Figure 2). In the first approach, we directly simulate gene genealogies for our samples and then overlay mutations on the simulated gene genealogy. Coalescent process is partitioned into two phases (before domestication where recombination happened freely and after domestication when recombination is greatly reduced due to selfing) [23]. For each focal genomic segment, we first simulated genealogical history for a non-recombining loci until we reach the time of domestication, then we approximate the coalescent process in the ancestral population by partitioning the focal segment into different sizes of non-recombining small segments (corresponding to different recombination rate in the wild population).

In order to explore a wider range of demographic histories, we employ the ms program [39] to simulate the evolution of genome sequences under both the independent and sequential domestication models (Figure 2). The demographic histories we explored include a range of values for population bottleneck and divergence time. The exact details of the simulations are presented in Text S1.

### Sequencing data

All the sequencing data from this study will be available at the FTP server hosted by Beijing Institute of Genomics (BIG), Chinese Academy of Sciences. Ftp address: ftp://ftp.big.ac.cn.

## Supporting Information

Figure S1 Genome-wide diversity as well as mean Fst values across the rice genome for three populations. The top panels show the diversity for three rice populations. Brown horizontal segments are overlapping LDRs identified in the current study. The bottom panels show the sliding window (100 kb window stepping at 10 kb) estimates of mean Fst values for three pair wise comparisons. Brown segments display the locations for the overlapping LDRs.
(PDF)

Figure S2 Fst distributions from real data as well as simulated demography for all sites. A) Observed cumulative plot for Fst between I and J; Fst distribution for overlapping LDRs are plotted in dashed lines. Solid lines are used for genome background. B) Observed cumulative plot for Fst between R and J. C) Simulated cumulative plot for Fst between I and J under an independent domestication history. D) Simulated cumulative plot for Fst between R and J under an independent domestication history. E) Simulated cumulative plot for Fst between I and J under a sequential domestication history. F) Simulated cumulative plot for Fst between R and J under a sequential domestication history. This is the same plot as Figure 4 in main text, but plotted for all sites rather than only sites where Fst(R, I)>0.5.
(PDF)

Table S1 Plant materials used in this study.
(DOC)

Table S2 Summary of sequencing data and reads mapping.
(DOC)

Table S3 θ per kb estimated from single platform or combined data. Only sites whose coverage in GA and SOLiD platform are both 6X or more are used. S is the number of segregating sites in a given region and S>1 counts the same sites but excludes singletons. S>2 excludes doubletons in addition. Estimates in the “Mocked” row do not distinguish GA and SOLiD reads and simply add up all reads. The numbers in this row show that sample sizes do not make the estimates lower. In contrast, the estimates in the “Combined” row take into consideration platform-dependent errors. Sample sizes between the two rows are comparable. Estimates of the “Literature” row were from Caicedo et al[20] and Tang et al[21]. Since japonica lines in our collection are all from the temperate zone, we used the corresponding number in the literature.
(DOC)

Table S4 Overlapping low diversity regions shared between *japonica* and *indica.* P value are testing the hypothesis whether Fst(I,J) is significantly shift to the left of Fst(R,J) or Fst(R,I) using one sided Kolmogorov-Smirnov test with R package (http://www.r-project.org/).
(DOC)

Table S5 Predicted candidate genes of domestication.
(DOC)

Text S1 Supporting methods and discussion.
(PDF)
