# Supplementary material for: Two Evolutionary Histories in the Genome of Rice: the Roles of Domestication Genes
Source: PLoS Genet. 2011 Jun 9;7(6):e1002100. doi: 10.1371/journal.pgen.1002100 (PMC3111475; doi:10.1371/journal.pgen.1002100)
Supplement: Figure S1 — Genome-wide diversity as well as mean Fst values across the rice genome for three populations. The top panels show the diversity for three rice populations. Brown horizontal segments are overlapping LDRs identified in the current study. The bottom panels show the sliding window (100 kb window stepping at 10 kb) estimates of mean Fst values for three pair wise comparisons. Brown segments display the locations for the overlapping LDRs. (PDF) [file pgen.1002100.s001.pdf]

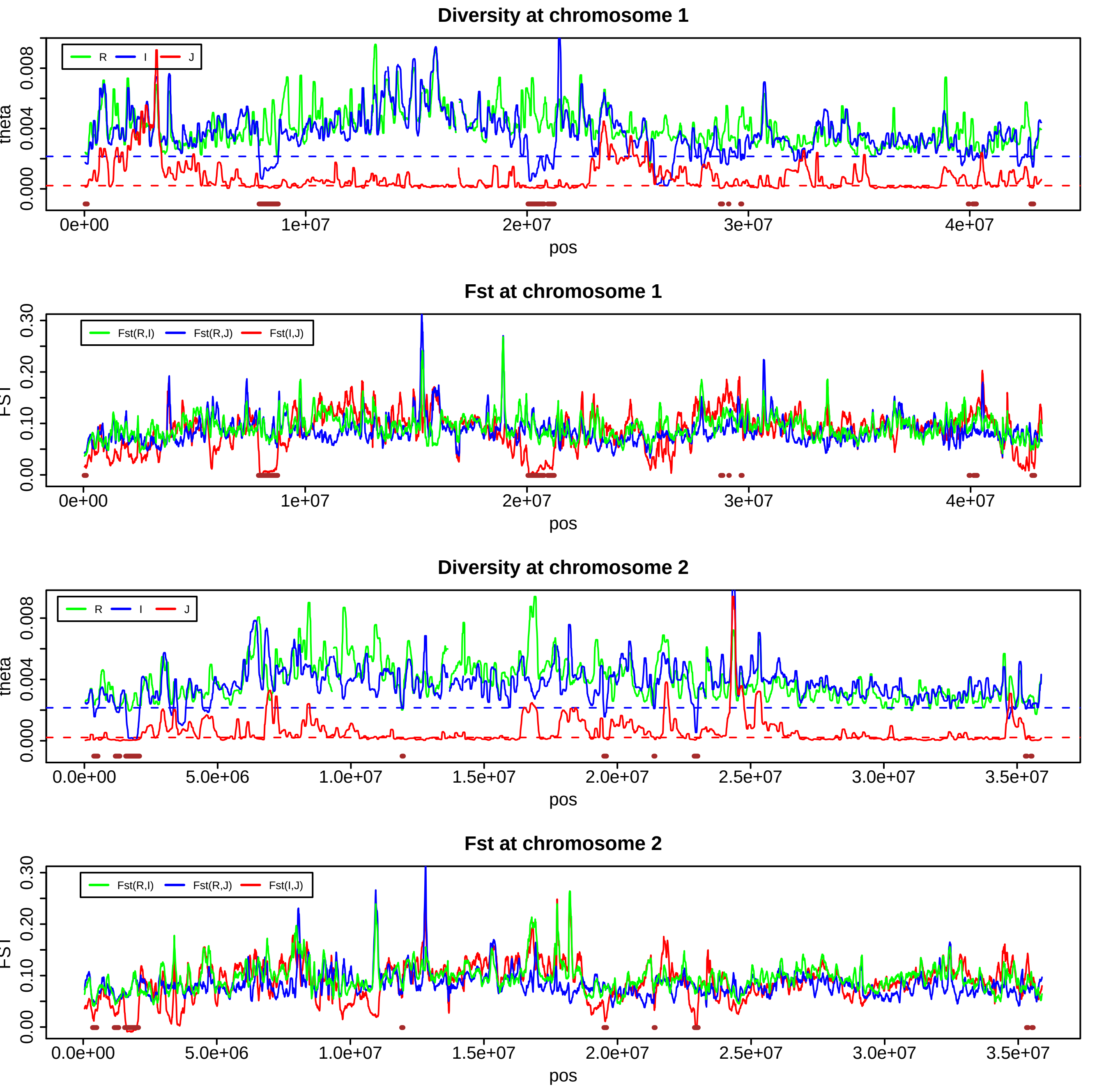

Diversity at chromosome 1
theta
R
I
J
0.000
0.004
0.008
0e+00
1e+07
2e+07
3e+07
4e+07
pos
Fst at chromosome 1
FST
Fst(R,I)
Fst(R,J)
Fst(I,J)
0.00
0.10
0.20
0.30
0e+00
1e+07
2e+07
3e+07
4e+07
pos
Diversity at chromosome 2
theta
R
I
J
0.000
0.004
0.008
0.0e+00
5.0e+06
1.0e+07
1.5e+07
2.0e+07
2.5e+07
3.0e+07
3.5e+07
pos
Fst at chromosome 2
FST
Fst(R,I)
Fst(R,J)
Fst(I,J)
0.00
0.10
0.20
0.30
0.0e+00
5.0e+06
1.0e+07
1.5e+07
2.0e+07
2.5e+07
3.0e+07
3.5e+07
pos

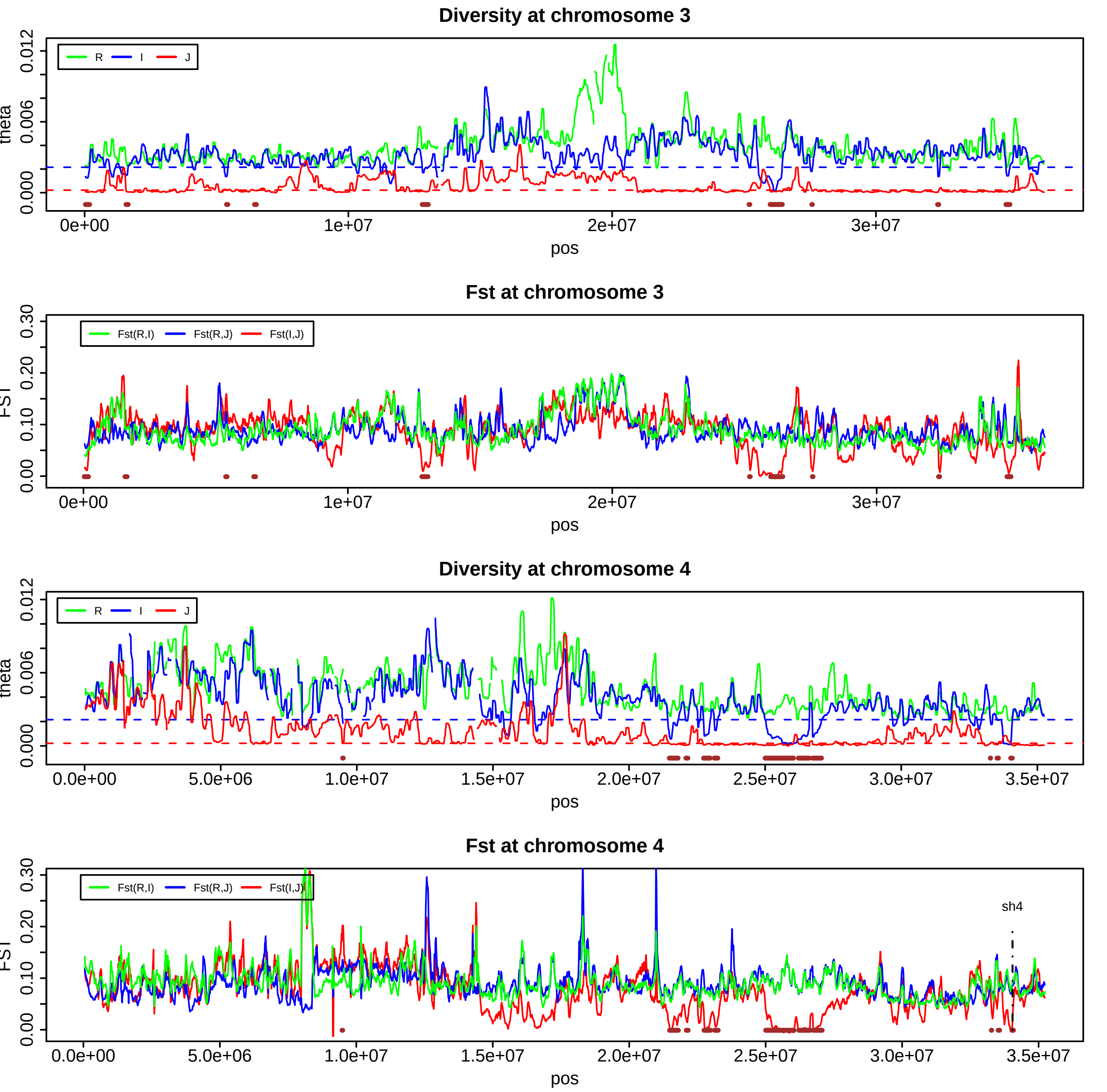
Diversity at chromosome 3
theta
R
I
J
0.000
0.006
0.012
0e+00
1e+07
2e+07
3e+07
pos
Fst at chromosome 3
FST
Fst(R,I)
Fst(R,J)
Fst(I,J)
0.00
0.10
0.20
0.30
0e+00
1e+07
2e+07
3e+07
pos
Diversity at chromosome 4
theta
R
I
J
0.000
0.006
0.012
0.0e+00
5.0e+06
1.0e+07
1.5e+07
2.0e+07
2.5e+07
3.0e+07
3.5e+07
pos
Fst at chromosome 4
FST
Fst(R,I)
Fst(R,J)
Fst(I,J)
sh4
0.00
0.10
0.20
0.30
0.0e+00
5.0e+06
1.0e+07
1.5e+07
2.0e+07
2.5e+07
3.0e+07
3.5e+07
pos

Diversity at chromosome 5

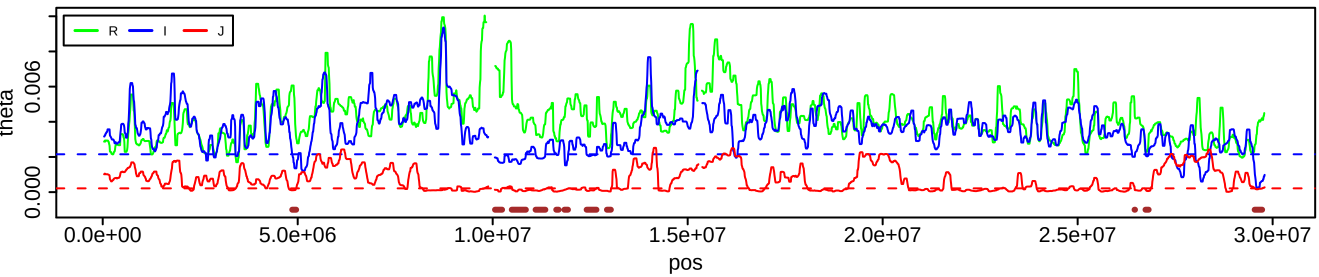

Fst at chromosome 5

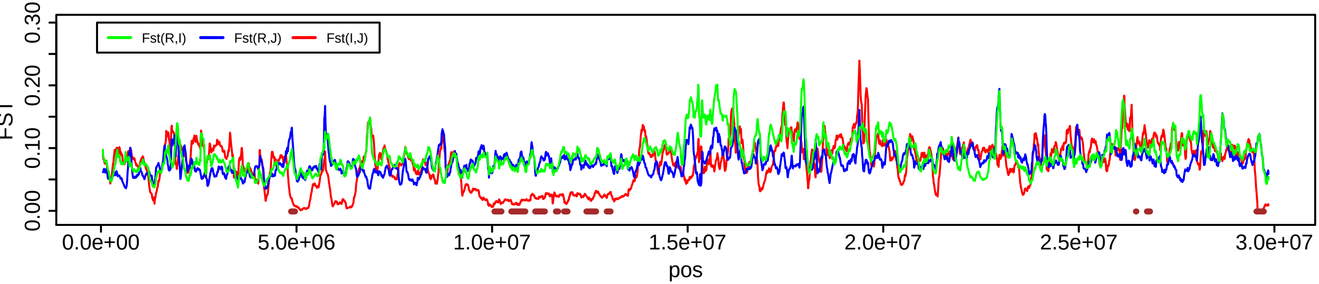

Diversity at chromosome 6

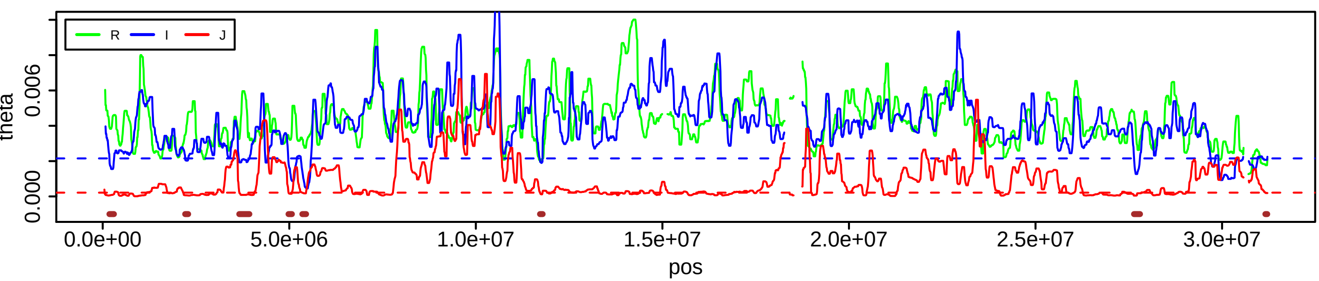

Fst at chromosome 6

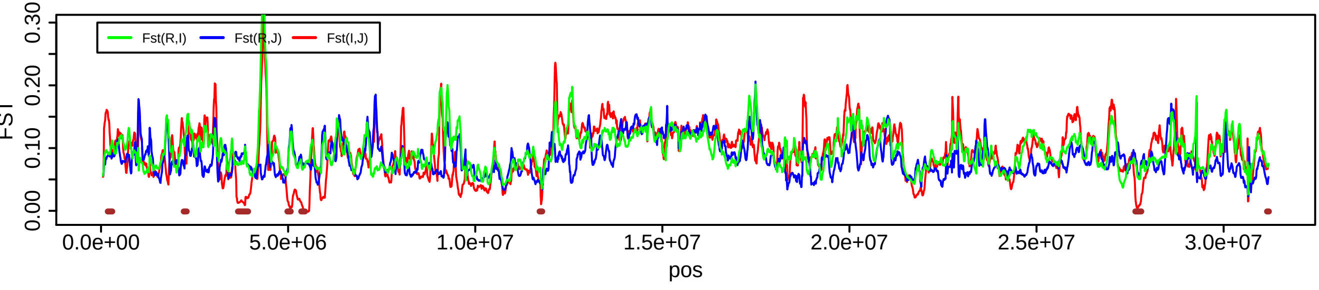

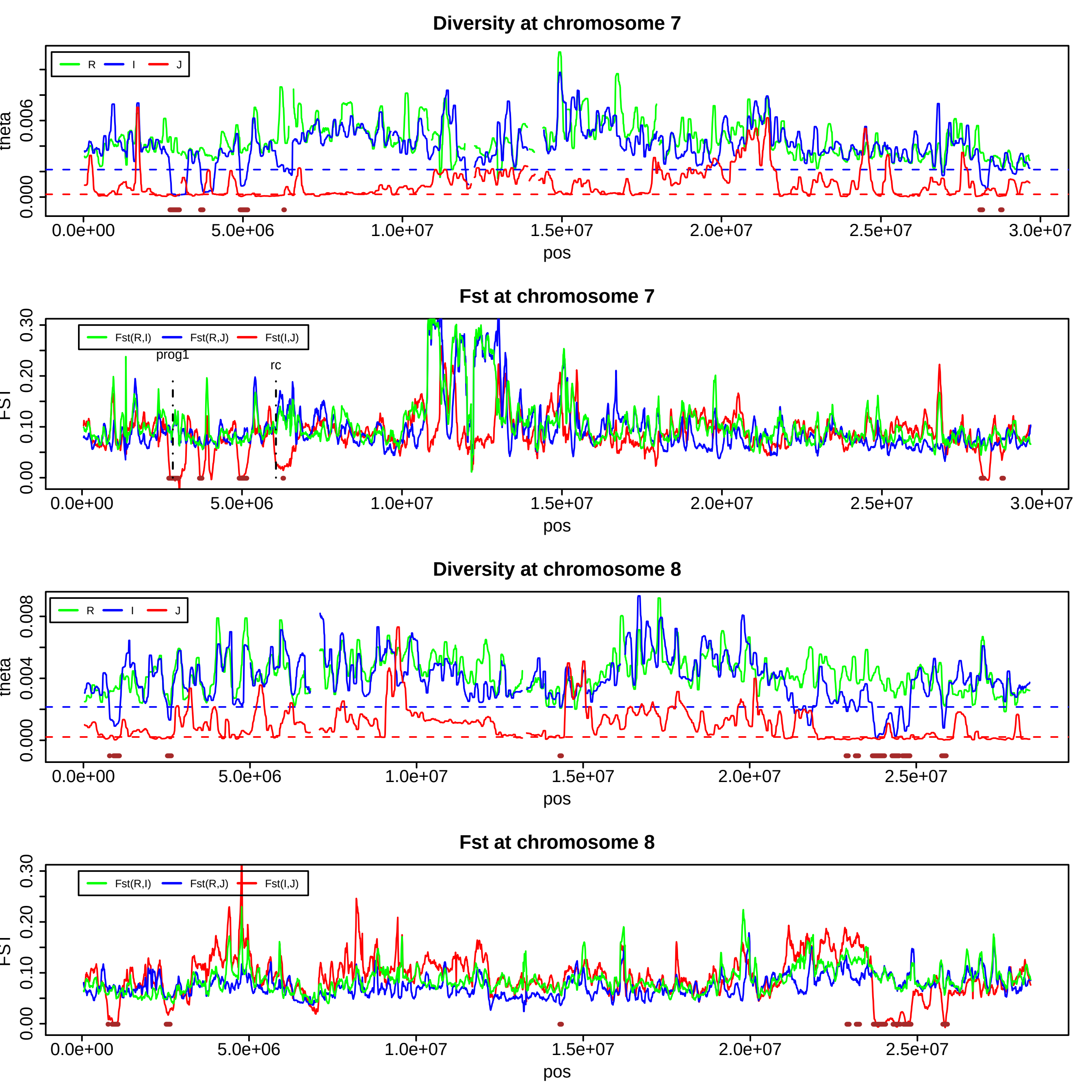

Diversity at chromosome 7
R
I
J
theta
0.000
0.006
0.0e+00
5.0e+06
1.0e+07
1.5e+07
2.0e+07
2.5e+07
3.0e+07
pos
Fst at chromosome 7
Fst(R,I)
Fst(R,J)
Fst(I,J)
prog1
rc
FST
0.00
0.10
0.20
0.30
0.0e+00
5.0e+06
1.0e+07
1.5e+07
2.0e+07
2.5e+07
3.0e+07
pos
Diversity at chromosome 8
R
I
J
theta
0.000
0.004
0.008
0.0e+00
5.0e+06
1.0e+07
1.5e+07
2.0e+07
2.5e+07
pos
Fst at chromosome 8
Fst(R,I)
Fst(R,J)
Fst(I,J)
FST
0.00
0.10
0.20
0.30
0.0e+00
5.0e+06
1.0e+07
1.5e+07
2.0e+07
2.5e+07
pos

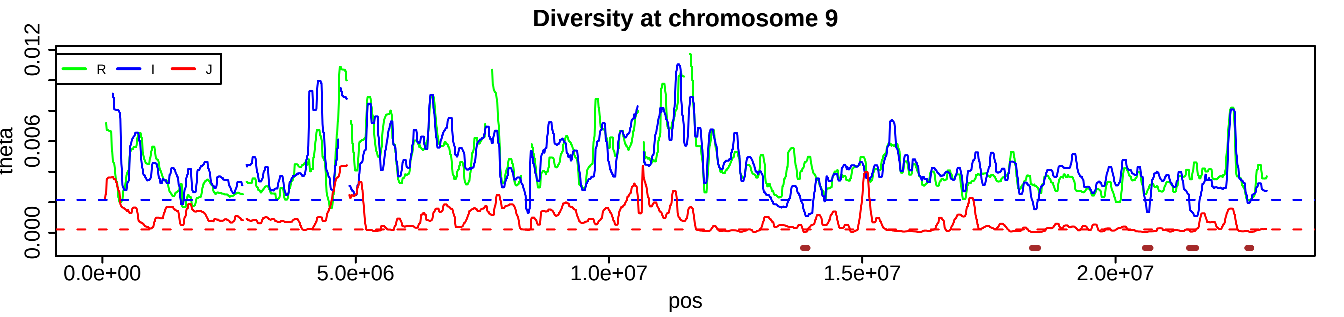

Diversity at chromosome 9
R
I
J
theta
0.000
0.006
0.012
0.0e+00
5.0e+06
1.0e+07
1.5e+07
2.0e+07
pos

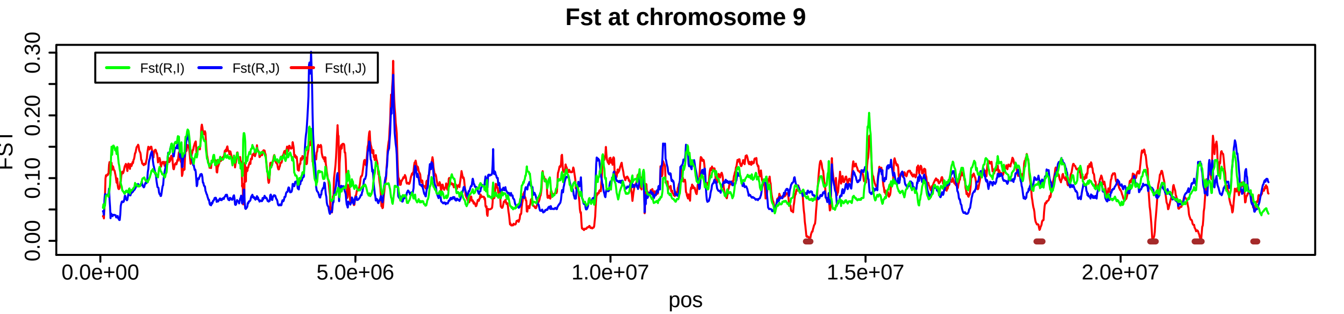

Fst at chromosome 9
Fst(R,I)
Fst(R,J)
Fst(I,J)
FST
0.00
0.10
0.20
0.30
0.0e+00
5.0e+06
1.0e+07
1.5e+07
2.0e+07
pos

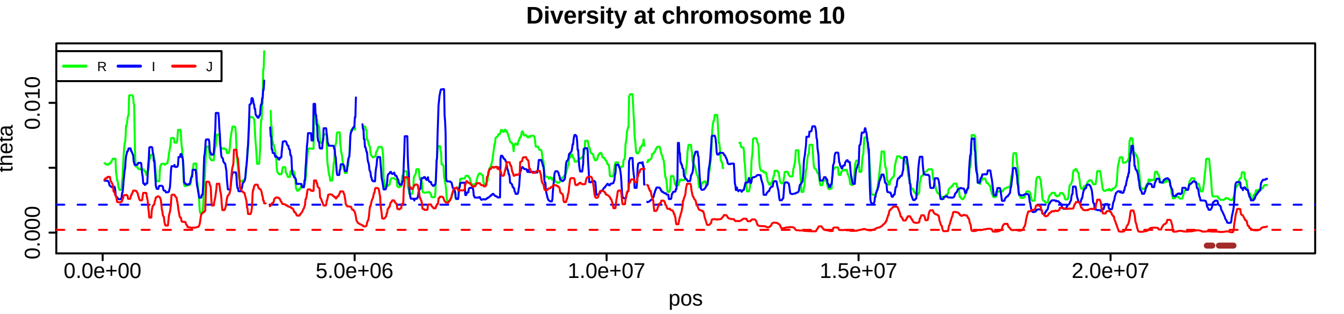

Diversity at chromosome 10
R
I
J
theta
0.000
0.010
0.0e+00
5.0e+06
1.0e+07
1.5e+07
2.0e+07
pos

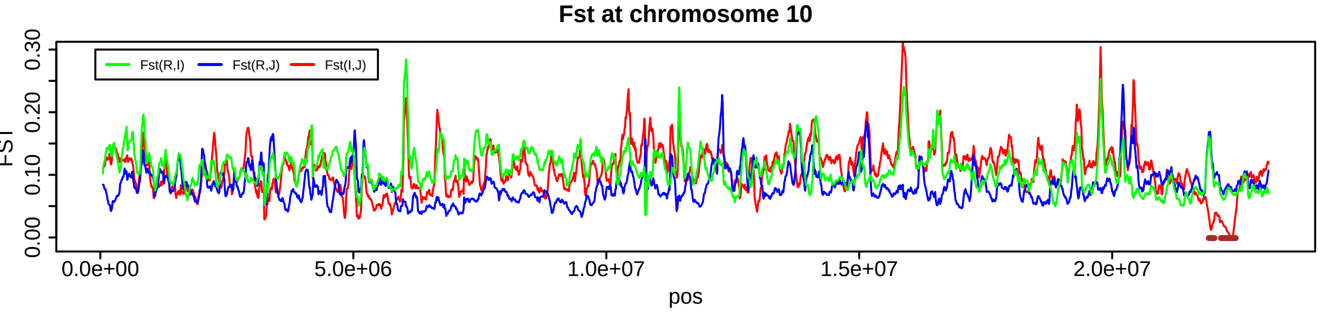

Fst at chromosome 10
Fst(R,I)
Fst(R,J)
Fst(I,J)
FST
0.00
0.10
0.20
0.30
0.0e+00
5.0e+06
1.0e+07
1.5e+07
2.0e+07
pos

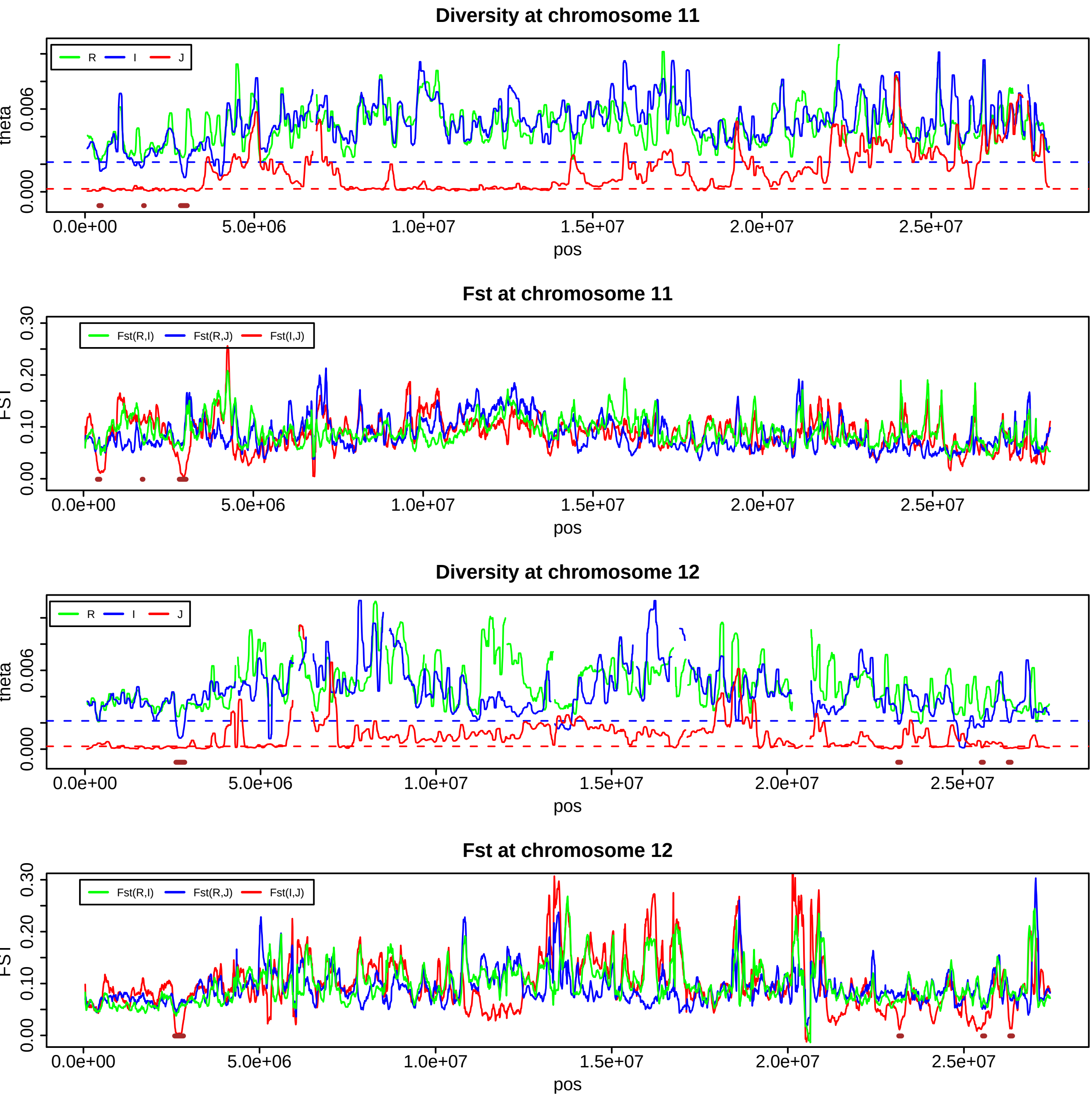

Diversity at chromosome 11
R
I
J
theta
0.000
0.006
0.0e+00
5.0e+06
1.0e+07
1.5e+07
2.0e+07
2.5e+07
pos
Fst at chromosome 11
Fst(R,I)
Fst(R,J)
Fst(I,J)
FST
0.00
0.10
0.20
0.30
0.0e+00
5.0e+06
1.0e+07
1.5e+07
2.0e+07
2.5e+07
pos
Diversity at chromosome 12
R
I
J
theta
0.000
0.006
0.0e+00
5.0e+06
1.0e+07
1.5e+07
2.0e+07
2.5e+07
pos
Fst at chromosome 12
Fst(R,I)
Fst(R,J)
Fst(I,J)
FST
0.00
0.10
0.20
0.30
0.0e+00
5.0e+06
1.0e+07
1.5e+07
2.0e+07
2.5e+07
pos
