# Supplementary figures and images for: Two Evolutionary Histories in the Genome of Rice: the Roles of Domestication Genes
Source: PLoS Genet. 2011 Jun 9;7(6):e1002100. doi: 10.1371/journal.pgen.1002100 (PMC3111475; doi:10.1371/journal.pgen.1002100)

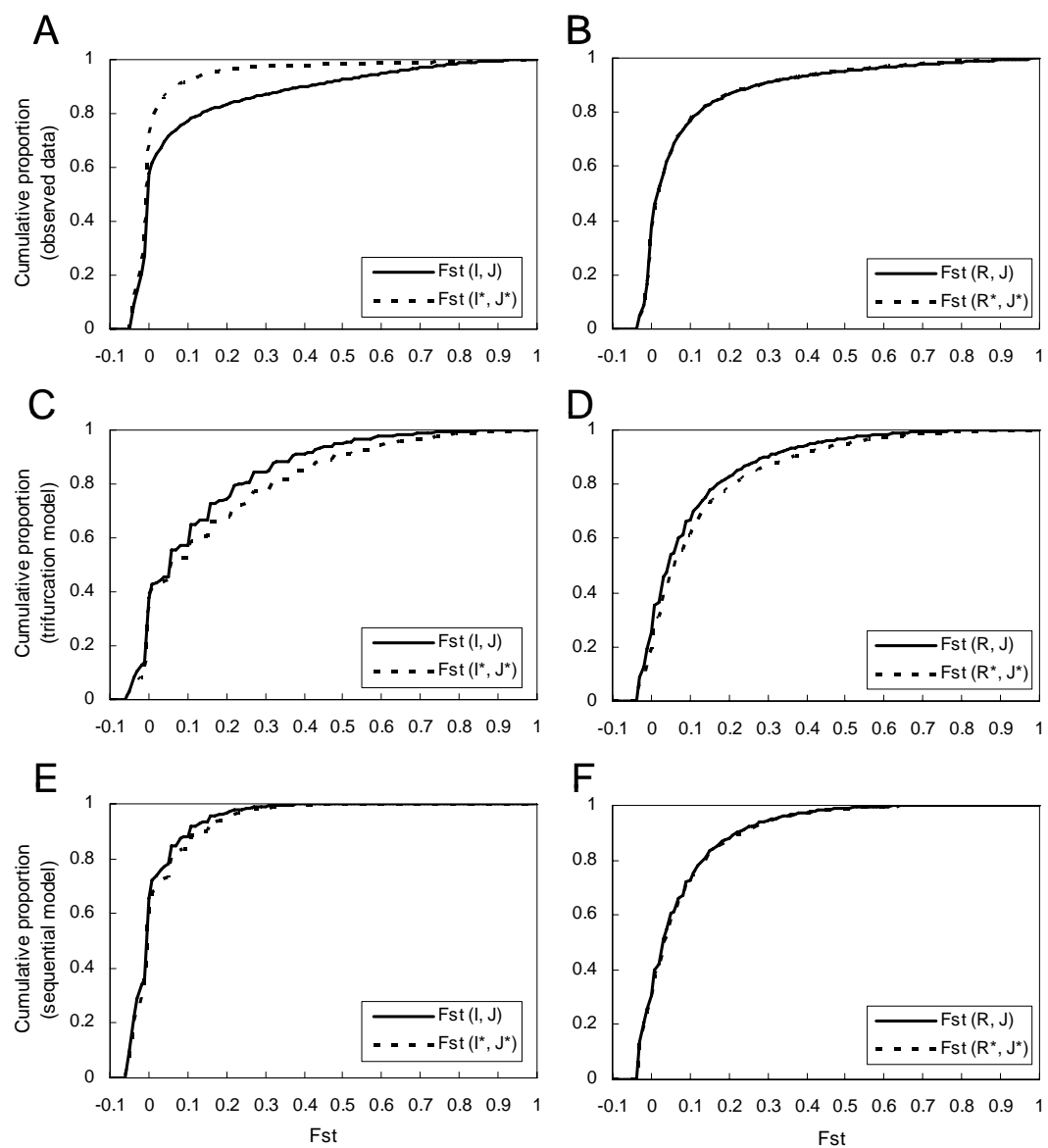

Supplement: Figure S2 — Fst distributions from real data as well as simulated demography for all sites. A) Observed cumulative plot for Fst between I and J; Fst distribution for overlapping LDRs are plotted in dashed lines. Solid lines are used for genome background. B) Observed cumulative plot for Fst between R and J. C) Simulated cumulative plot for Fst between I and J under an independent domestication history. D) Simulated cumulative plot for Fst between R and J under an independent domestication history. E) Simulated cumulative plot for Fst between I and J under a sequential domestication history. F) Simulated cumulative plot for Fst between R and J under a sequential domestication history. This is the same plot as Figure 4 in main text, but plotted for all sites rather than only sites where Fst(R, I)>0.5. (PDF) [file pgen.1002100.s002.pdf]
