# Supplementary material for: Two Evolutionary Histories in the Genome of Rice: the Roles of Domestication Genes
Source: PLoS Genet. 2011 Jun 9;7(6):e1002100. doi: 10.1371/journal.pgen.1002100 (PMC3111475; doi:10.1371/journal.pgen.1002100)
Supplement: Table S1 — Plant materials used in this study. (DOC) [file pgen.1002100.s003.doc]

**Table S1. Plant materials used in this study.**

| Taxa | IRRI # | Origin |
| --- | --- | --- |
| *O. sativa* ssp. *japonica* | 418 | JAPAN |
| *O. sativa* ssp. *japonica* | 1107 | CHINA |
| *O. sativa* ssp. *japonica* | 1468 | CHINA |
| *O. sativa* ssp. *japonica* | **1479** | CHINA |
| *O. sativa* ssp. *japonica* | 2545 | JAPAN |
| *O. sativa* ssp. *japonica* | 2856 | TAIWAN |
| *O. sativa* ssp. *japonica* | 2975 | TAIWAN |
| *O. sativa* ssp. *japonica* | 3826 | PHILIPPINES |
| *O. sativa* ssp. *japonica* | 3830 | PHILIPPINES |
| *O. sativa* ssp. *japonica* | 3845 | PHILIPPINES |
| *O. sativa* ssp. *japonica* | 5295 | JAPAN |
| *O. sativa* ssp. *japonica* | 7396 | JAPAN |
| *O. sativa* ssp. *japonica* | 8191 | JAPAN |
| *O. sativa* ssp. *japonica* | 12731 | JAPAN |
| *O. sativa* ssp. *japonica* | 34300 | CHINA |
| *O. sativa* ssp. *japonica* | 40252 | JAPAN |
| *O. sativa* ssp. *japonica* | 42576 | TAIWAN |
| *O. sativa* ssp. *japonica* | 55471 | SOUTH KOREA |
| *O. sativa* ssp. *japonica* | 55530 | SOUTH KOREA |
| *O. sativa* ssp. *japonica* | 58286 | AFGHANISTAN |
| *O. sativa* ssp. *japonica* | 61882 | SOUTH KOREA |
| *O. sativa* ssp. *indica* | 1708 | UNITED STATES |
| *O. sativa* ssp. *indica* | 3969 | PHILIPPINES |
| *O. sativa* ssp. *indica* | 5803 | THAILAND |
| *O. sativa* ssp. *indica* | **6663** | INDIA |
| *O. sativa* ssp. *indica* | **7755** | SRI LANKA |
| *O. sativa* ssp. *indica* | 8240 | TAIWAN |
| *O. sativa* ssp. *indica* | **8952** | SRI LANKA |
| *O. sativa* ssp. *indica* | 9432 | JAPAN |
| *O. sativa* ssp. *indica* | 9804 | PHILIPPINES |
| *O. sativa* ssp. *indica* | 26872 | PHILIPPINES |
| *O. sativa* ssp. *indica* | **27513** | BANGLADESH |
| *O. sativa* ssp. *indica* | 27748 | THAILAND |
| *O. sativa* ssp. *indica* | 43369 | INDONESIA |
| *O. sativa* ssp. *indica* | 43545 | INDONESIA |
| *O. sativa* ssp. *indica* | 45011 | INDIA |
| *O. sativa* ssp. *indica* | 46202 | INDIA |
| *O. sativa* ssp. *indica* | 51250 | CHINA |
| *O. sativa* ssp. *indica* | 51400 | CHINA |
| *O. sativa* ssp. *indica* | 56036 | VIETNAM |
| *O. sativa* ssp. *indica* | 58930 | NEPAL |
| *O. sativa* ssp. *indica* | 66970 | PHILIPPINES |
| *O. sativa* ssp. *indica* | 73994 | CHINA |
| *O. rufipogon* | 82040 | THAILAND |
| *O. rufipogon* | 86451 | MYANMAR |
| *O. rufipogon* | 86476 | INDIA |
| *O. rufipogon* | 88788 | BANGLADESH |
| *O. rufipogon* | 100678 | TAIWAN |
| *O. rufipogon* | 102186 | INDIA |
| *O. rufipogon* | 103305 | PHILIPPINES |
| *O. rufipogon* | 103308 | TAIWAN |
| *O. rufipogon* | 103404 | BANGLADESH |
| *O. rufipogon* | 104803 | THAILAND |
| *O. rufipogon* | 105491 | MALAYSIA |
| *O. rufipogon* | 105698 | NEPAL |
| *O. rufipogon* | 105709 | INDIA |
| *O. rufipogon* | 105720 | CAMBODIA |
| *O. rufipogon* | 105767 | THAILAND |
| *O. rufipogon* | 105888 | BANGLADESH |
| *O. rufipogon* | 105889 | BANGLADESH |
| *O. rufipogon* | 105910 | THAILAND |
| *O. rufipogon* | 105942 | THAILAND |
| *O. rufipogon* | 106161 | LAOS |
| *O. rufipogon* | 106288 | PAPUA NEW GUINEA |
| *O. rufipogon* | 106505 | PAPUA NEW GUINEA |
| *O. rufipogon* | DX60^*^ | CHINA |

***** An accession of *O. rufipogon* collected from Jiangxi Province of China.

Those accession numbers in red are cultivars with red pericarps.
