# Supplementary material for: Two Evolutionary Histories in the Genome of Rice: the Roles of Domestication Genes
Source: PLoS Genet. 2011 Jun 9;7(6):e1002100. doi: 10.1371/journal.pgen.1002100 (PMC3111475; doi:10.1371/journal.pgen.1002100)
Supplement: Table S2 — Summary of sequencing data and reads mapping. (DOC) [file pgen.1002100.s004.doc]

**Table S**2. Summary of sequencing data and reads mapping.

| **Platform** | **Taxa** | **Total number of reads** | **Unique mapping rate** | **Effective data*** | **Average depth** |
| --- | --- | --- | --- | --- | --- |
|  | ***japonica*** | 191926898 | 67.3% | 5.48 | 14.7 |
| **GA** | ***indica*** | 182487212 | 56.1% | 4.35 | 11.7 |
|  | ***rufipogon*** | 172181612 | 47.8% | 3.44 | 9.2 |
|  |  |  |  |  |  |
|  | ***japonica*** | 606302031 | 37.2% | 8.46 | 22.7 |
| **SOLiD** | ***indica*** | 547136763 | 28.2% | 6.05 | 16.2 |
|  | ***rufipogon*** | 539238113 | 22.7% | 4.77 | 12.8 |

*Discarded the low quality bases in the data of which phred scores are lower than 15
