# Supplementary material for: Two Evolutionary Histories in the Genome of Rice: the Roles of Domestication Genes
Source: PLoS Genet. 2011 Jun 9;7(6):e1002100. doi: 10.1371/journal.pgen.1002100 (PMC3111475; doi:10.1371/journal.pgen.1002100)
Supplement: Table S3 — θ per kb estimated from single platform or combined data. Only sites whose coverage in GA and SOLiD platform are both 6X or more are used. S is the number of segregating sites in a given region and S>1 counts the same sites but excludes singletons. S>2 excludes doubletons in addition. Estimates in the “Mocked” row do not distinguish GA and SOLiD reads and simply add up all reads. The numbers in this row show that sample sizes do not make the estimates lower. In contrast, the estimates in the “Combined” row take into consideration platform-dependent errors. Sample sizes between the two rows are comparable. Estimates of the “Literature” row were from Caicedo et al[20] and Tang et al[21]. Since japonica lines in our collection are all from the temperate zone, we used the corresponding number in the literature. (DOC) [file pgen.1002100.s005.doc]

**Table S**3. θ per kb estimated from single platform or combined data.

| **Platform** | **Data used** | ***japonica*** | ***indica*** | ***O. rufipogon*** |
| --- | --- | --- | --- | --- |
| **GA (Solexa)** | **S(All sites)** | 8.55 | 10.13 | 11.53 |
|  | **S_>1_** | 1.44 | 5.05 | 6.01 |
|  | **S_>2_** | 1.13 | 4.70 | 5.30 |
|  |  |  |  |  |
| **SOLiD** | **S(All sites)** | 13.89 | 13.98 | 12.46 |
|  | **S_>1_** | 3.09 | 5.30 | 5.77 |
|  | **S_>2_** | 1.64 | 4.24 | 4.64 |
|  |  |  |  |  |
| **Mocked** | **S(All sites)** | 17.68 | 17.34 | 16.24 |
|  | **S_>1_** | 3.10 | 6.07 | 6.93 |
|  | **S_>2_** | 1.77 | 4.96 | 5.71 |
|  |  |  |  |  |
| **Combined** | **S(All sites)** | 1.28 | 4.02 | 4.77 |
|  | **S_>1_** | 0.90 | 3.72 | 4.04 |
|  | **S_>2_** | 0.75 | 3.47 | 3.59 |
|  |  |  |  |  |
| **Literature** |  | 0.69 ~ 1.10 | 1.58 ~ 2.74 | 3.70 ~ 5.83 |

Only sites whose coverage in GA and SOLiD platform are both 6X or more are used. S is the number of segregating sites in a given region and S>1 counts the same sites but excludes singletons. S>2 excludes doubletons in addition. Estimates in the “Mocked” row do not distinguish GA and SOLiD reads and simply add up all reads. The numbers in this row show that sample sizes do not make the estimates lower. In contrast, the estimates in the “Combined” row take into consideration platform-dependent errors. Sample sizes between the two rows are comparable. Estimates of the “Literature” row were from Caicedo *et al*[20] and Tang *et al*[21]. Since *japonica* lines in our collection are all from the temperate zone, we used the corresponding number in the literature.
