# Supplementary material for: Two Evolutionary Histories in the Genome of Rice: the Roles of Domestication Genes
Source: PLoS Genet. 2011 Jun 9;7(6):e1002100. doi: 10.1371/journal.pgen.1002100 (PMC3111475; doi:10.1371/journal.pgen.1002100)
Supplement: Table S4 — Overlapping low diversity regions shared between japonica and indica. P value are testing the hypothesis whether Fst(I,J) is significantly shift to the left of Fst(R,J) or Fst(R,I) using one sided Kolmogorov-Smirnov test with R package (http://www.r-project.org/). (DOC) [file pgen.1002100.s006.doc]

***Table S4. Overlapping low diversity regions shared between japonica and indica.***

| **Chromosome** | **Start(kb)** | **Length(kb)** | **P value(KS test)** |
| --- | --- | --- | --- |
| chr01 | 30 | 100 | 0.26 |
| chr01 | 7890 | 870 | 1.39e-228 |
| chr01 | 20040 | 560 | 7.03e-139 |
| chr01 | 20650 | 120 | 1.15e-17 |
| chr01 | 20930 | 300 | 2.28e-59 |
| chr01 | 28730 | 100 | 0.99 |
| chr01 | 29100 | 20 | 1 |
| chr01 | 29650 | 50 | 0.46 |
| chr01 | 39930 | 50 | 1 |
| chr01 | 40130 | 170 | 0.99 |
| chr01 | 42760 | 130 | 6.92e-17 |
| chr02 | 350 | 160 | 0.67 |
| chr02 | 1160 | 170 | 0.0008 |
| chr02 | 1550 | 520 | 6.64e-146 |
| chr02 | 11920 | 50 | 0.85 |
| chr02 | 19490 | 100 | 1.89e-22 |
| chr02 | 21370 | 40 | 1 |
| chr02 | 22880 | 140 | 3.98e-31 |
| chr02 | 35310 | 60 | 0.99 |
| chr02 | 35510 | 50 | 0.90 |
| chr03 | 30 | 160 | 0.00018 |
| chr03 | 1570 | 80 | 0.99 |
| chr03 | 5380 | 50 | 1 |
| chr03 | 6440 | 70 | 1 |
| chr03 | 12800 | 230 | 5.89e-79 |
| chr03 | 25190 | 30 | 2.06e-09 |
| chr03 | 25990 | 460 | 6.83e-107 |
| chr03 | 27570 | 20 | 0.018 |
| chr03 | 32340 | 40 | 0.0017 |
| chr03 | 34930 | 150 | 1.61e-16 |
| chr04 | 9480 | 20 | 1 |
| chr04 | 21480 | 320 | 1.94e-60 |
| chr04 | 22090 | 70 | 7.55e-30 |
| chr04 | 22740 | 240 | 1.94e-65 |
| chr04 | 23130 | 130 | 2.33e-36 |
| chr04 | 25000 | 1040 | 0 |
| chr04 | 26220 | 400 | 2.22e-185 |
| chr04 | 26740 | 330 | 6.17e-135 |
| chr04 | 33270 | 10 | 0.0025 |
| chr04 | 33520 | 50 | 0.911 |
| chr04^a^ | 34020 | 60 | 2.97e-26 |
| chr05 | 4860 | 100 | 3.14e-17 |
| chr05 | 10060 | 180 | 1.23e-14 |
| chr05 | 10490 | 360 | 1.47e-36 |
| chr05 | 11100 | 250 | 1.65e-12 |
| chr05 | 11630 | 60 | 3.41e-05 |
| chr05 | 11840 | 90 | 9.07e-14 |
| chr05 | 12410 | 250 | 3.50e-32 |
| chr05 | 12930 | 100 | 8.58e-19 |
| chr05 | 26460 | 10 | 0.14 |
| chr05 | 26740 | 80 | 0.83 |
| chr05 | 29540 | 190 | 6.89e-89 |
| chr06 | 180 | 120 | 0.99 |
| chr06 | 2210 | 80 | 0.85 |
| chr06 | 3660 | 270 | 4.79e-32 |
| chr06 | 4980 | 90 | 8.89e-47 |
| chr06 | 5350 | 100 | 2.66e-16 |
| chr06 | 11720 | 70 | 1 |
| chr06 | 27640 | 160 | 8.49e-15 |
| chr06 | 31160 | 50 | 0.96 |
| chr07^b^ | 2710 | 300 | 4.16e-81 |
| chr07 | 3670 | 80 | 1.07e-30 |
| chr07 | 4910 | 240 | 4.71e-68 |
| chr07­^c^ | 6280 | 20 | 2.06e-09 |
| chr07 | 28100 | 90 | 4.15e-18 |
| chr07 | 28750 | 50 | 0.44 |
| chr08 | 780 | 10 | 0.61 |
| chr08 | 900 | 180 | 2.07e-26 |
| chr08 | 2520 | 120 | 0.05 |
| chr08 | 14300 | 50 | 1 |
| chr08 | 22890 | 70 | 1 |
| chr08 | 23170 | 100 | 1 |
| chr08 | 23680 | 370 | 9.01e-186 |
| chr08 | 24270 | 220 | 3.69e-64 |
| chr08 | 24580 | 230 | 4.31e-95 |
| chr08 | 25760 | 140 | 1.07e-31 |
| chr09 | 13830 | 90 | 1.27e-14 |
| chr09 | 18350 | 120 | 5.66e-38 |
| chr09 | 20580 | 110 | 3.48e-57 |
| chr09 | 21450 | 140 | 2.50e-63 |
| chr09 | 22600 | 80 | 1 |
| chr10 | 21910 | 110 | 4.04e-61 |
| chr10 | 22150 | 290 | 3.58e-71 |
| chr11 | 410 | 70 | 9.36e-14 |
| chr11 | 1730 | 20 | 1 |
| chr11 | 2820 | 200 | 2.03e-39 |
| chr12 | 2590 | 250 | 1.07e-21 |
| chr12 | 23150 | 80 | 4.549e-05 |
| chr12 | 25530 | 60 | 0.023 |
| chr12 | 26300 | 80 | 4.54e-05 |

^a^: sh4 gene is located in this segment; ^b^: PROG1 is in this region ^c^: Rc gene is close to this LDR.

P value are testing the hypothesis whether Fst(I,J) is significantly shift to the left of Fst(R,J) or Fst(R,I) using one sided Kolmogorov-Smirnov test with R package ( http://www.r-project.org/).
