# Supplementary material for: Two Evolutionary Histories in the Genome of Rice: the Roles of Domestication Genes
Source: PLoS Genet. 2011 Jun 9;7(6):e1002100. doi: 10.1371/journal.pgen.1002100 (PMC3111475; doi:10.1371/journal.pgen.1002100)
Supplement: Table S5 — Predicted candidate genes of domestication. (DOC) [file pgen.1002100.s007.doc]

**Table S5. Predicted candidate genes of domestication.**

| **# genes**  **per segment *** | **Locus** | **Chr** | **Functional Annotation** |
| --- | --- | --- | --- |
| 1 | LOC_Os01g36640 | chr01 | disease resistance protein RPM1, putative, expressed |
| 1^a^ | LOC_Os01g14220 | chr01 | expressed protein |
| 1 | LOC_Os02g38020 | chr02 | inorganic phosphate transporter 2-1, chloroplast precursor, putative, expressed |
| 1 | LOC_Os03g44710 | chr03 | YABBY domain containing protein, putative, expressed |
| 1 | LOC_Os04g38590 | chr04 | hypothetical protein |
| 1 | LOC_Os04g57510 | chr04 | exostosin family domain containing protein, expressed |
| 1 | LOC_Os05g20290 | chr05 | retrotransposon protein, putative, Ty3-gypsy subclass |
| 1 | LOC_Os06g09910 | chr06 | phosphopantothenoylcysteine decarboxylase, putative, expressed |
| 1 | LOC_Os06g10450 | chr06 | expressed protein |
| 1 | LOC_Os07g47090 | chr07 | KIP1, putative, expressed |
| 1 | LOC_Os08g40710 | chr08 | retrotransposon protein, putative, unclassified, expressed |
| 1 | LOC_Os09g30280 | chr09 | glycosyl transferase 8 domain containing protein, putative, expressed |
| 1 | LOC_Os09g37400 | chr09 | OsSAUR45 - Auxin-responsive SAUR gene family member |

* Criteria for picking the candidate genes are presented in the main text. a: gene with relatively higher diversity in a larger study.
