# Supplementary material for: Two Evolutionary Histories in the Genome of Rice: the Roles of Domestication Genes
Source: PLoS Genet. 2011 Jun 9;7(6):e1002100. doi: 10.1371/journal.pgen.1002100 (PMC3111475; doi:10.1371/journal.pgen.1002100)
Supplement: Text S1 — Supporting methods and discussion. (PDF) [file pgen.1002100.s008.pdf]

Text S1

A. Coalescent simulations

A.1 Demographic history. We first simulated gene genealogies of samples from multiple populations and then sprinkled mutations on the genealogy via the Poisson process, with means proportional to branch lengths. We assume *O. rufipogon* to be an equilibrium population with a stable effective population size, N_R. In the framework of coalescent theory, the average coalescence time of *O. rufipogon* to the most recent common ancestor S is $4N_R$ generations (T). Three groups estimated the MRCA of *indica* and *japonica* to be between 0.2 and 0.4 million years ago[1-3]. Assuming one generation per year, one would suggest N_R to be about 75,000 (300,000 / 4). Caicedo *et al* also estimated N_R to be 57,471~118,110[4]. Given the θ (= $4N\mu$) estimates of Table 1, μ can then be obtained.

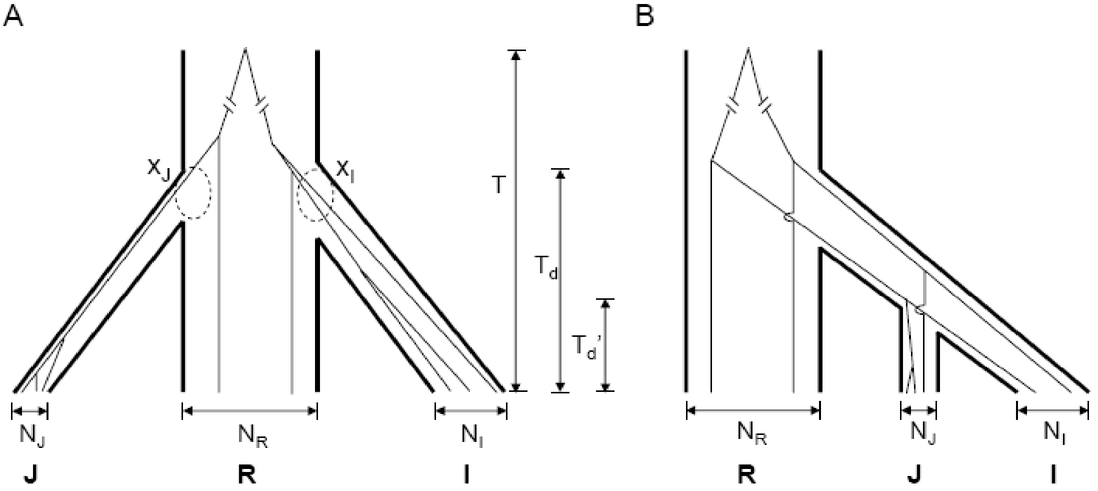

Figure S3. A cartoon version of the demographic history (same as Figure 3).

For *indica* and *japonica*, coalescence proceeds in two stages. The first stage is the time of domestication, up to T_d before present, during which the effective population size is N_I and N_J, respectively. T_d has been estimated to be about 6000 years[5]. The ratio of T_d/T is about 0.02. With the genome-wide diversity of each taxon shown in Table 1 (main text), it is then possible to estimate N_I and N_J. In simulations, we used the published values of 0.27 and 0.12 for N_I/N_R and N_J/N_R respectively[5], which are close to our own estimates. The second stage for *indica* and *japonica* is coalescence prior to T_d. The process in this stage is the same as it is in *O. rufipogon*.

A.2 Recombination. We are interested in the means (of θ and Fst, for example), which are not affected by recombination. However, recombination will affect the correlations between nearby segments, and thus will influence the variance of diversity. In addition, because domesticated rice is largely a selfing species where recombination is greatly reduced, we approximate recombination reduction by simulating the genealogy of non-recombining segments, which range from 5 to 500 kb. For example, when the

window size is 100 kb and the non-recombining segment is set at 20 kb, the simulated θ for the 100 kb segment would be the sum of the values of the five smaller segments.

For *rufipogon*, we used N_R to simulate coalescent events from the present to MRCA. For *japonica*, we used N_J (N_I for *indica*) to simulate coalescent events from the present to time T_d, and N_R to simulate coalescent events from time T_d until all lineages coalesced to MRCA. For each non-recombining segment, we simulated a genealogical tree and calculate $S_{segment}$. We then added up multiple $S_{segment}$ to obtain S_{window}.

B. Numbers of LDRs in cultivars can be compatible with neutral demography and selfing

In the analysis of LDRs, the distribution of θ matters and recombination has to be considered. Our objective is to see if the distribution of θ for large DNA segments may be sufficiently broad due to population bottlenecks and self-pollination alone, yielding high proportions of LDRs reported in Table 2.

B.1 Obtain LDR cutoff. Similar to what we did in analyzing real data, we identified the cutoff of LDRs using 1kb units to shuffle the simulated genomes, generating 100 units for each 100 kb window. For each 1kb segment, we simulated a genealogical tree and counted its total branch length (T_{br}). Given a mutation rate μ, the number of segregating sites (S_{unit}) was Poisson distributed with the mean $T_{br}\mu$. We then summed up the 100 S_{unit} to obtain the 100 kb-wide S_{window}. We recorded the lowest S_{window} in all 4000 windows for a 400 Mb simulated genome, denoted S_{min}. By shuffling the genome 200 times, we selected the 10th smallest S_{min} as the low cutoff. The cutoff is defined as the level at which 95% of the simulations do not yield even one 100 kb segment in the entire genome (the same as cutoffs used in real data).

B.2 Different levels of recombination. We approximate recombination reduction by simulating the genealogy of non-recombining segments, which range from 5 to 500 kb. For example, when the window size is 200 kb and the non-recombining segment is set at 20 kb, the simulated θ for the 200 kb segment would be the sum of the values of the ten smaller segments.

Table S6. Percentages of genome that are LDRs under selfing conditions and demography.

Size of non-recombining segments in simulations	Fraction of genome (%)			
	japonica (N_J=0.12N_R)*	***japonica*** (N_J=0.06N_R)	***indica*** (N_I=0.27N_R)*	***O. rufipogon***
20 kb	0.26	2.18	0.04	0.01
50 kb	2.44	8.19	0.41	0.04

100 kb	6.66	13.82	2.05	0.19
200 kb	11.38	24.72	5.60	0.92
Observed+	26.38		6.15	0.25

*: used values in the simulations; +: proportion of segments greater than 200kb in Table 2 main text

Table S6 summarizes results from simulations with different levels of recombination and population bottlenecks. From Table S6, we can see that 1) cultivars have more LDRs than the wild population, matching our observations in Table 2 (main text); 2) the proportion of the genome that is in LDRs can vary over a wide range depending on recombination rate (size of the non-recombining segments); and 3) the size of the population bottleneck can have dramatic effects on the proportion of LDRs (for example, if we halve the size of the bottleneck in *japonica* (from 0.12 to 0.06), the observed proportion of LDRs can increase by at least two fold). In summary, both demography and selfing (recombination rate) in cultivars are likely to generate observed proportions of LDRs.

We also did coalescent simulations using ms[6] program with similar set of parameters. Especially we modified ms program to have different recombination rate before and after domestication. The results we observed from the ms simulation are very similar to Table S6 (data not shown).

LDR cutoffs generated using the shuffling method reflect genome-wide lower bounds of diversity we expect to see if genetic variation between adjacent genomic segments is independent. Population bottlenecks and selfing are likely to create higher correlation between nearby positions, thus increasing variances in diversity across genome. The proportion of the genome below this cutoff is also an indication of the variance of genetic diversity.

The levels of recombination in different rice populations vary widely. For example, linkage disequilibrium in *japonica* is the largest and can range between 150kb to more than 500kb. In *indica*, LD extends over shorter distances (~75kb). In wild populations, LD is quite short and typically spans less than 40kb[7]. The levels of recombination presented in Table S6 were chosen to cover this range of recombination rates.

Although we are making the argument that levels of LDRs in different rice populations can be compatible with non-selective forces (demography and selfing), we are not ruling out selection as a competitive explanation. It is widely recognized that demography and selection often leave similar traces. Thus, LDRs identified in this study comprise a mixture of segments some of which were may have been generated through the action of natural selection.

C. Fst distributions within LDRs under different demographic histories

Both cultivars are self-pollinators whereas *O. rufipogon* is largely an outcrossing species. To understand the joint influences of demography and selfing, we carried out coalescence simulations to match demographic and recombination parameters with the observed patterns of LDRs (i.e., the size distribution of Table 2 and the overall genetic diversity of Table 1). The genealogies were simulated according to either an independent domestication model of Figure 3A or a sequential domestication model of Figure 3B.

In the model of Figure 3A, we assume that *O. rufipogon* is an equilibrium population with an effective size of N_R, which has been estimated at about 75,000[4]. At time T_d in the past (estimated to be about 6000 years ago)[5], *indica* and *japonica* were domesticated. Domestication happened separately with a reduced population size of N_I for *indica* and N_J for *japonica*. Effective recombination was lowered as a result of selfing in both cultivars.

Because sites in LDRs have lower x_I (number of ancestral lineages from *indica* that still exist at time T_d ago) and x_J (number of ancestral lineages from *japonica* that still exist at time T_d ago), we can use the values of x_I and x_J to identify LDRs. The values of x_I and x_J are variable within certain ranges. We chose the values of x_I and x_J which made the proportion of total sites closest to the values in Table 2 (31.28% for *japonica* and 8.9% for *indica*). For example, in independent domestication model ($T_d/T=0.02$), the proportion of $x_J<5$ is about 30% and the proportion of $x_J<6$ is about 58%. Therefore we choose 1~4 as the value of x_J in LDRs.

We ran the simulations under the independent domestication and sequential domestication models and obtained distributions of Fst. We randomly selected 1 million sites which were polymorphic in the pooled data of three populations (excluding sites that were singletons in only one species) and with depths between 20X and 40X in the *rufipogon* data. We used these allele frequencies in *rufipogon* as the distribution at T_d. We used 0.02 as the ratio of T_d/T in Figure 4. In the independent domestication model, we simulated coalescent events from the present to time T_d in each taxon for each site. We recorded the number of lineages left at time T_d for *japonica* and *indica* (x_I and x_J). We recorded the genealogy and randomly generated allele frequency at present in each taxon using the genealogy and the allele frequency at T_d. In the sequential domestication model, we first simulated coalescent events from present to time T_d' for *indica* and *japonica*, respectively. We recorded the number of lineages left at time T_d' for *japonica* and *indica*, respectively (x_I and x_J). We then treated *japonica* and *indica* as a single population and simulated until time T_d. We used ratio of $T_{d'}/T_d$ at 0.1 for simulating Figure 4(main text).

D. Similar distributions of Fst values within LDRs under a single demographic history

The coalescent simulations implemented in the previous sections (Figure S3) are relatively simple. We wanted to explore a wider range of demographic scenarios. Since there is already standard coalescent packages that can simulate these more complicated demographic histories, we didn't implement the coalescent process ourselves as in the

previous sections. We instead used the ms package to simulate the rice demographic history.

Our question is: if there is a single demographic history genome-wide, can we observe large differences between Fst distributions depicted in Figure 4A and 4B?

D.1 Demographic history. We implemented a demographic history based on modification of the methods described in Caicedo et al 2007[4] to broadly match the diversity patterns in the three rice genomes (Figure S4 and S5).

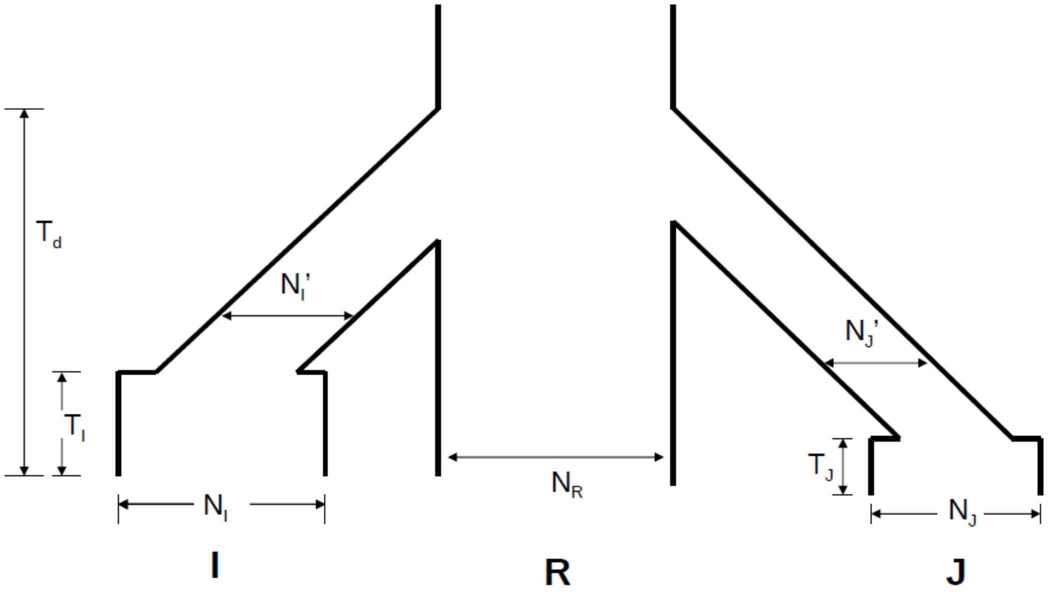

Figure S4. Cartoon of an independent demographic history for rice populations.

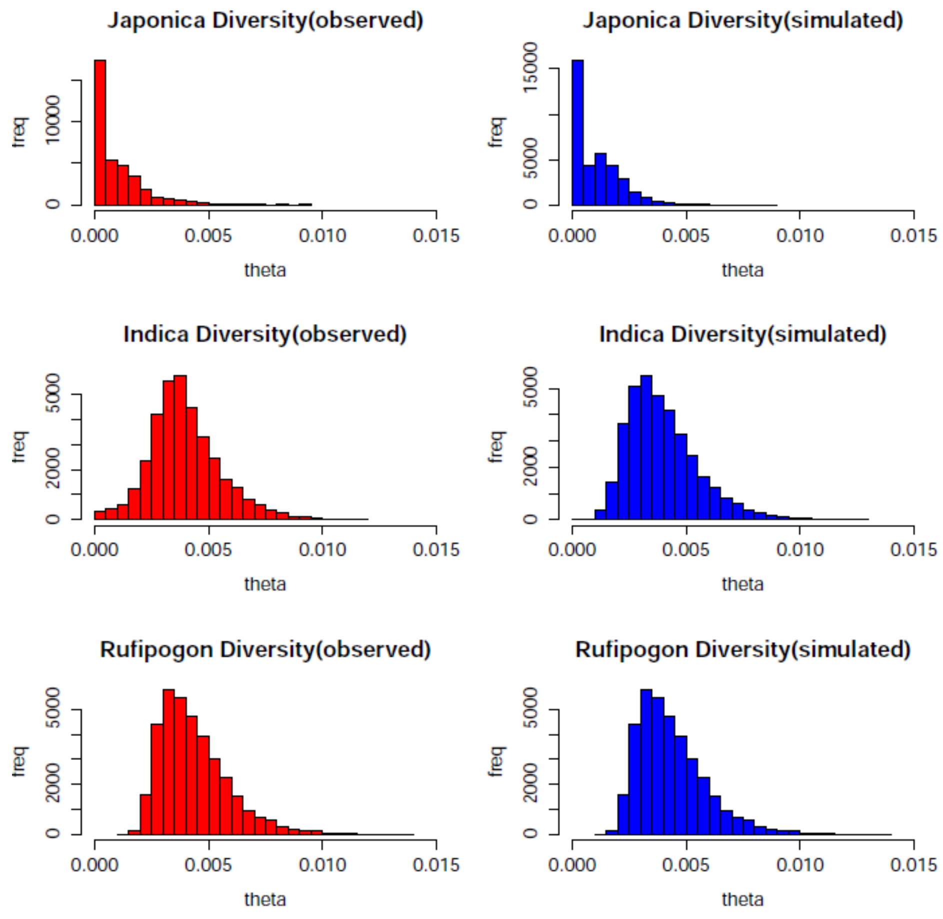

Figure S5. Theta distributions for three populations.

The final parameters and the ms code for simulating 20 haplotypes from *indica* (pop1), 20 haplotypes from *japonica* (pop2) and 1 sample from *rufipogon* (pop3) are: ms 41 1 -t theta -r 0.01*theta sites -I 3 20 20 1 7.000000 -en 0.012000 1 0.330000 -en 0.008500 2 0.005500 -ej 0.020000 1 3 -ej 0.020000 2 3 -em 0.020000 3 1 0 -em 0.020000 3 2 0 -n 1 0.500000 -n 2 0.120000

D.2 Measurement in Fst distributions

To measure distances between two cumulative distributions, we define a distance measure D(f1,f2) as the maximal difference between f1(x) and f2(x), i.e. max_x [f1(x)-f2(x)], where f1(x) and f2(x) are two cumulative distributions. If f1 is never higher than f2(x) (i.e. to the right or underneath of f2, for example Figure S6A), then D(f1,f2) is zero, while D(f2,f1) will be greater than zero (indicated in Figure S6A).

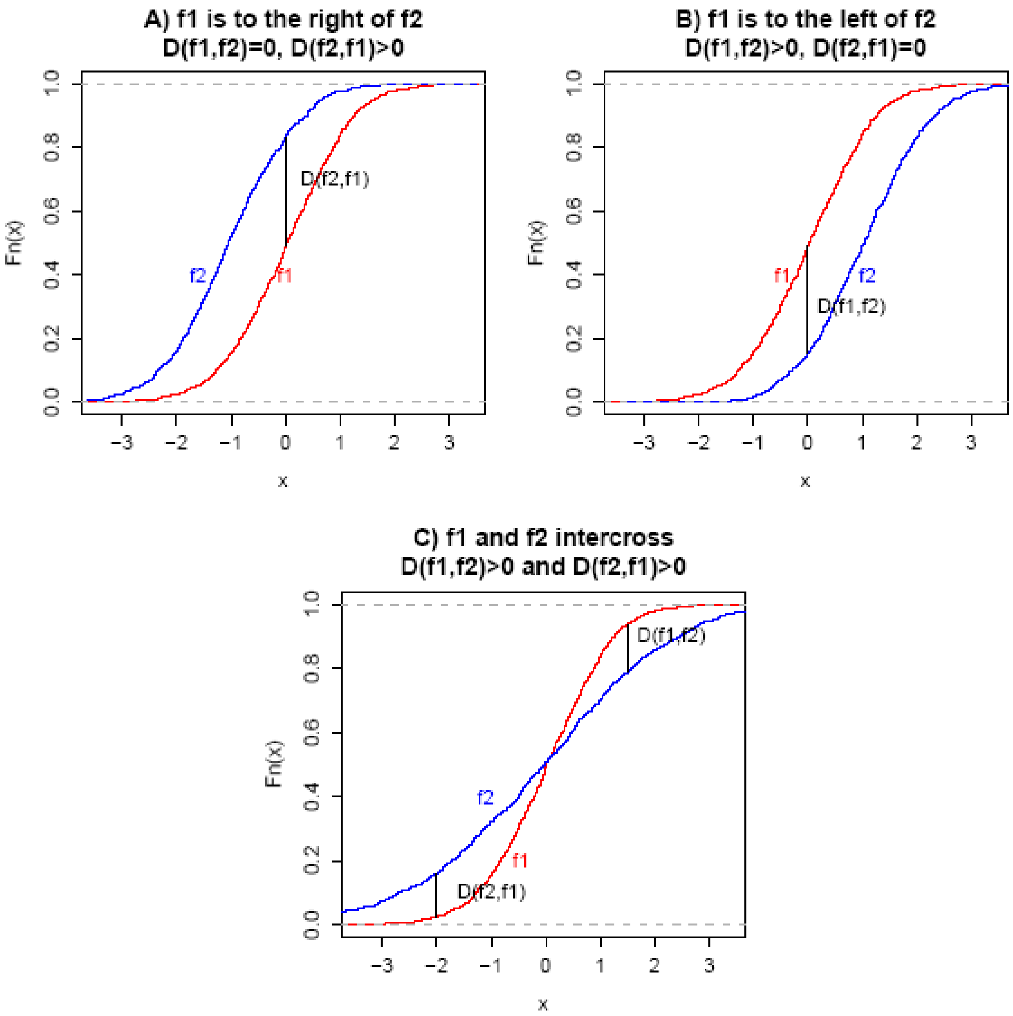

Figure S6. Cartoon examples of two cumulative distributions.

The value of D reflects the maximal departure between two distributions in a certain direction depending the order of f1 and f2. If the two distributions cross with each other (Figure S6C), then both D(f1,f2) and D(f2,f1) will be greater than zero. Thus the joint value of D(f1,f2) and D(f2,f1) captures the relative relationship between two distributions. If two distributions are close to each other, we expect D(f1,f2) and D(f2,f1) to be very small and in a two-dimensional plot very close to (0,0).

Fst distributions for the genomic background (between I and J) and overlapping LDRs (between I* and J*) in Figure 4A (main text) are quite disparate. The same is true for Fst between *rufipogon* and *japonica* (Figure 4B, main text). We want to investigate whether the observed discrepancies in Fst distributions between genomic background and overlapping LDRs are compatible with some single neutral demographic histories.

In order to systematically investigate the variation in demographic scenarios, we chose to vary one parameter at a time from a known demographic history and check the behavior of Fst distributions at each value (Figure S4). The exact parameter values are

listed in Table S8.

We explored eight demographic histories in our simulations (Table S7 and Figure S4). While our exploration of parameter space is not exhaustive, it should give us a sense whether demographic history can generate large differences between Fst distributions i.e. from the genome background vs overlapping LDRs.

Table S7. Different demographic scenarios explored in the current study.

Parameter*	Parameter_Before	Parameter_After	Case ID	Note
Default			0	default demography
N_I	0.5	0.7	1	bigger pop size for *indica*
T_I	0.012	0.006	2	longer bottleneck for *indica*
N_I’	0.33	0.22	3	stronger bottleneck for *indica*
N_J	0.12	0.24	4	bigger pop size for *japonica*
T_J	0.0085	0.016	5	shorter bottleneck for *japonica*
N_J’	0.0055	0.01	6	weaker bottleneck for *japonica*
T_d	0.02	0.04	7	longer divergence time

* see Figure S4

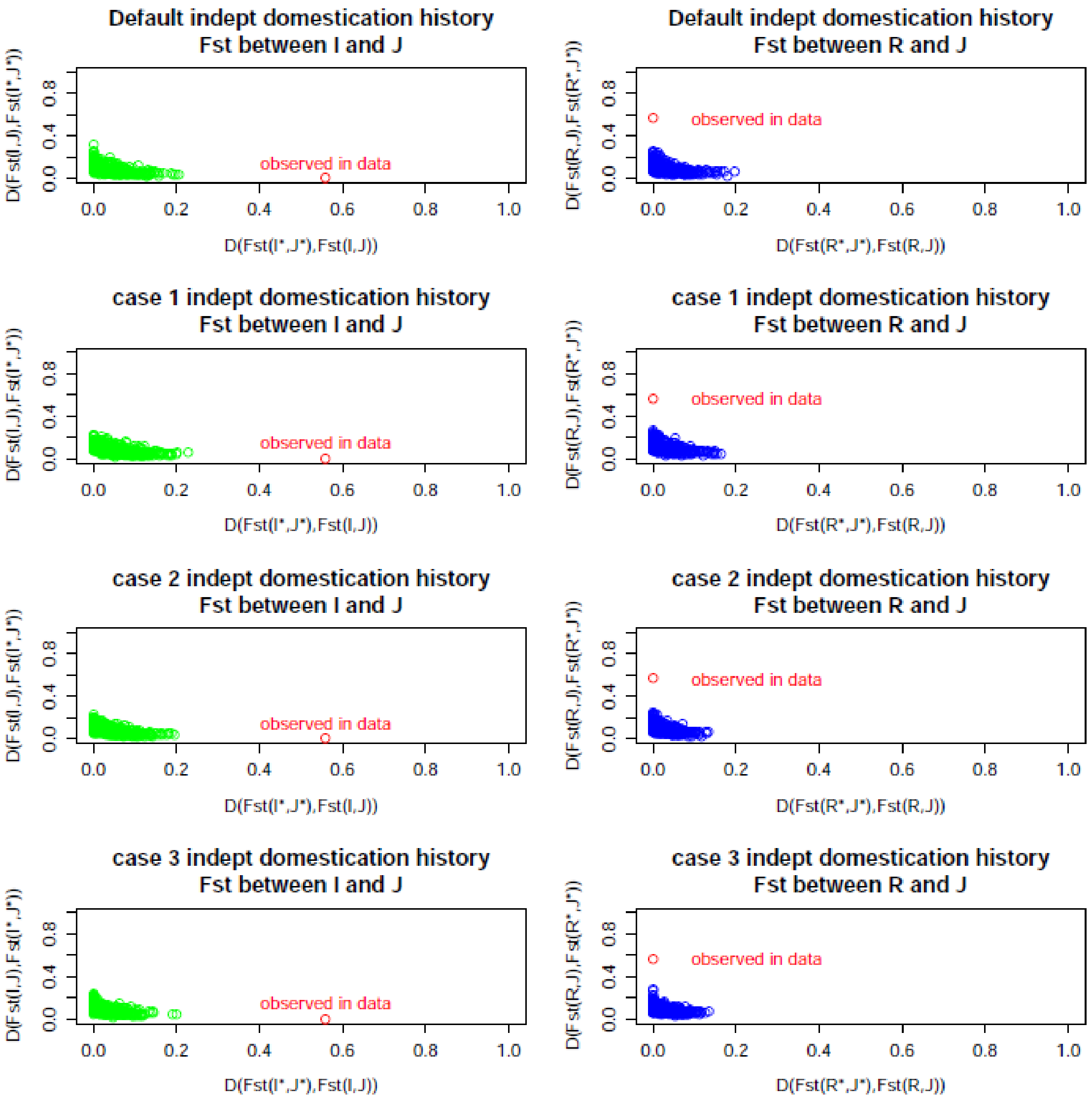

Default indept domestication history
Fst between I and J
D(Fst(I,J),Fst(I*,J*))
D(Fst(I*,J*),Fst(I,J))
observed in data
Default indept domestication history
Fst between R and J
D(Fst(R,J),Fst(R*,J*))
D(Fst(R*,J*),Fst(R,J))
observed in data
case 1 indept domestication history
Fst between I and J
observed in data
case 1 indept domestication history
Fst between R and J
observed in data
case 2 indept domestication history
Fst between I and J
observed in data
case 2 indept domestication history
Fst between R and J
observed in data
case 3 indept domestication history
Fst between I and J
observed in data
case 3 indept domestication history
Fst between R and J
observed in data

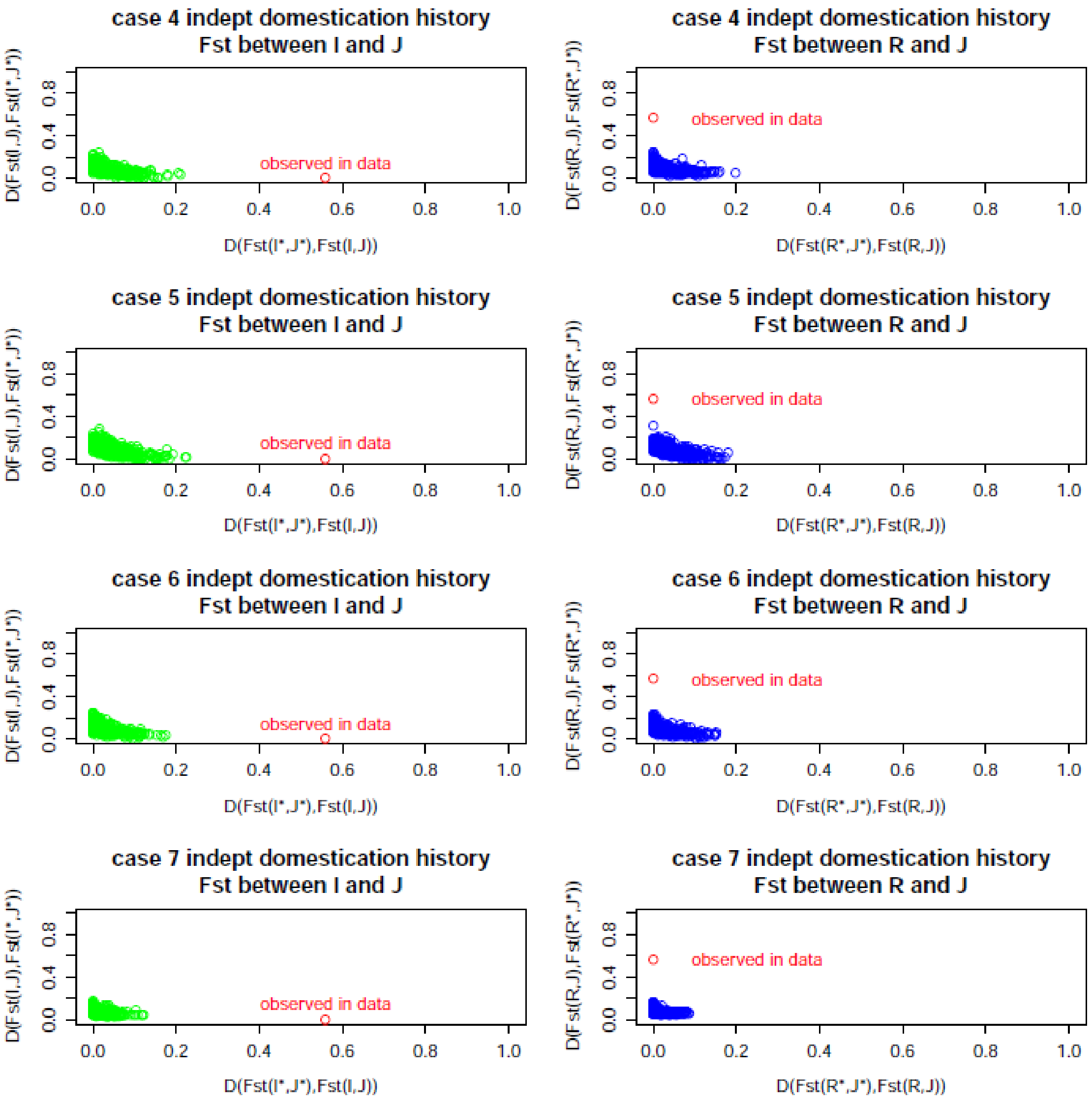

Figure S7. Distances in Fst distributions between genomic background and overlapping LDRs for different independent domestication histories. Overlapping LDRs are defined by identifying the same proportion of the genome as the LDRs as observed in real data.

In Figure S7, we plot D values between cumulative Fst distributions in the genomic background and the overlapping LDRs for both I,J and R,J. Similar to the analyses of real data, we focused on sites with appreciable population differentiation (Fst(R,I) > 0.5, also see main text). Each demographic scenario corresponds to two panels (I vs J and R vs J). It is clear from Figure S7 that under all the demographic scenarios we explored, if the genomic background and overlapping LDRs are generated from the same demographic history, the observed distances between them are always quite small as compared with what is observed in real data (shown as a red dot). Thus, observed differences in Fst distributions between genomic background and overlapping LDR in Figure 4 (main text)

are quite unlikely to be compatible with a single demographic history.

We also simulated a sequential demographic history similar to independent history illustrated above. The domestication history is depicted in Figure S8 and Table S8.

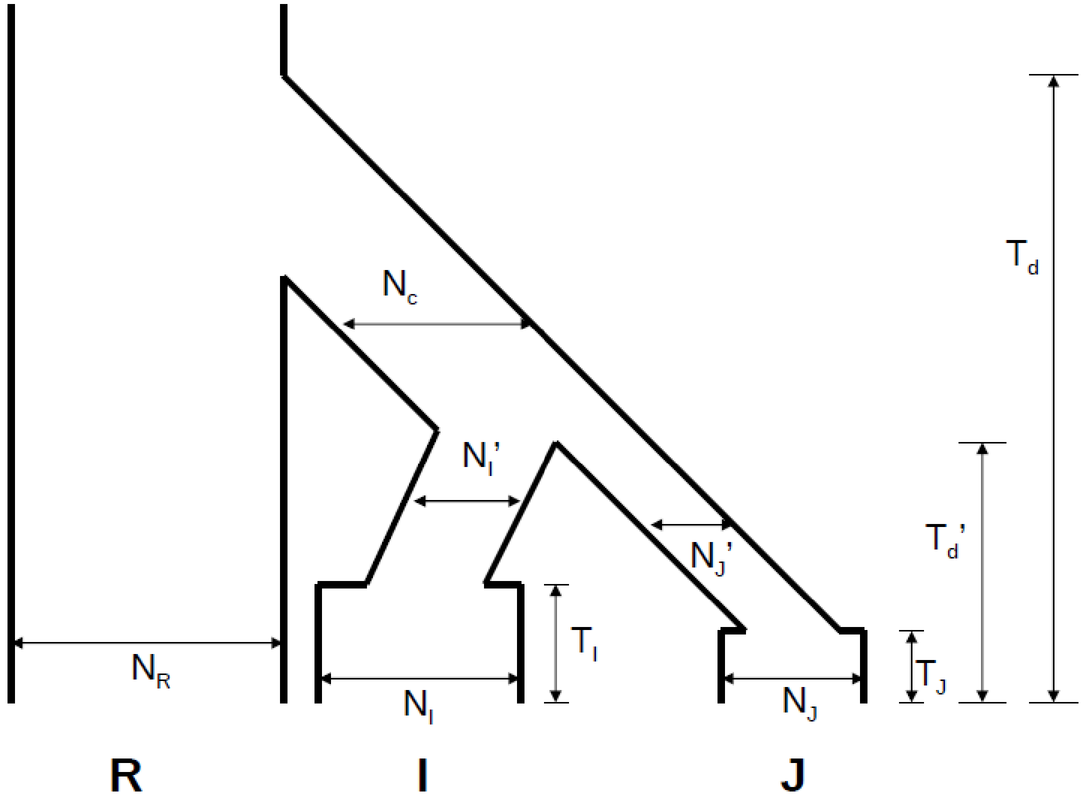

Figure S8. Cartoon of a sequential demographic history for rice populations.

Table S8. Different demographic scenarios explored in the current study.

Parameter*	Parameter_Before	Parameter_After	Case ID	Note
default			0	default demography
N_I	0.5	0.7	1	bigger pop size for *indica*
T_I	0.012	0.006	2	longer bottleneck for *indica*
N_I'	0.33	0.22	3	stronger bottleneck for *indica*
N_J	0.12	0.24	4	bigger pop size for *japonica*
T_J	0.0085	0.016	5	shorter bottleneck for *japonica*
N_J'	0.0055	0.01	6	weaker bottleneck for *japonica*
T_d	0.02	0.04	7	longer divergence time
T_d'	0.016	0.018	8	Deeper divergence time for (I,J)
N_c	0.5	0.8	9	Bigger ancestral population size for (I,J)

*see Figure S8

The distances between Fst distributions are shown in Figure S9. Similar to what is observed in Figure S7, the differences are much smaller in the simulations than what we observed in real data (Figure 4A and 4B). Therefore, the differences in Fst distributions are indicating mosaic evolutionary processes affecting two different parts of the genome. In particular, Fst distributions in overlapping LDRs shift toward the sequential domestication model whereas the bulk of the genome follows the independent domestication model (main text).

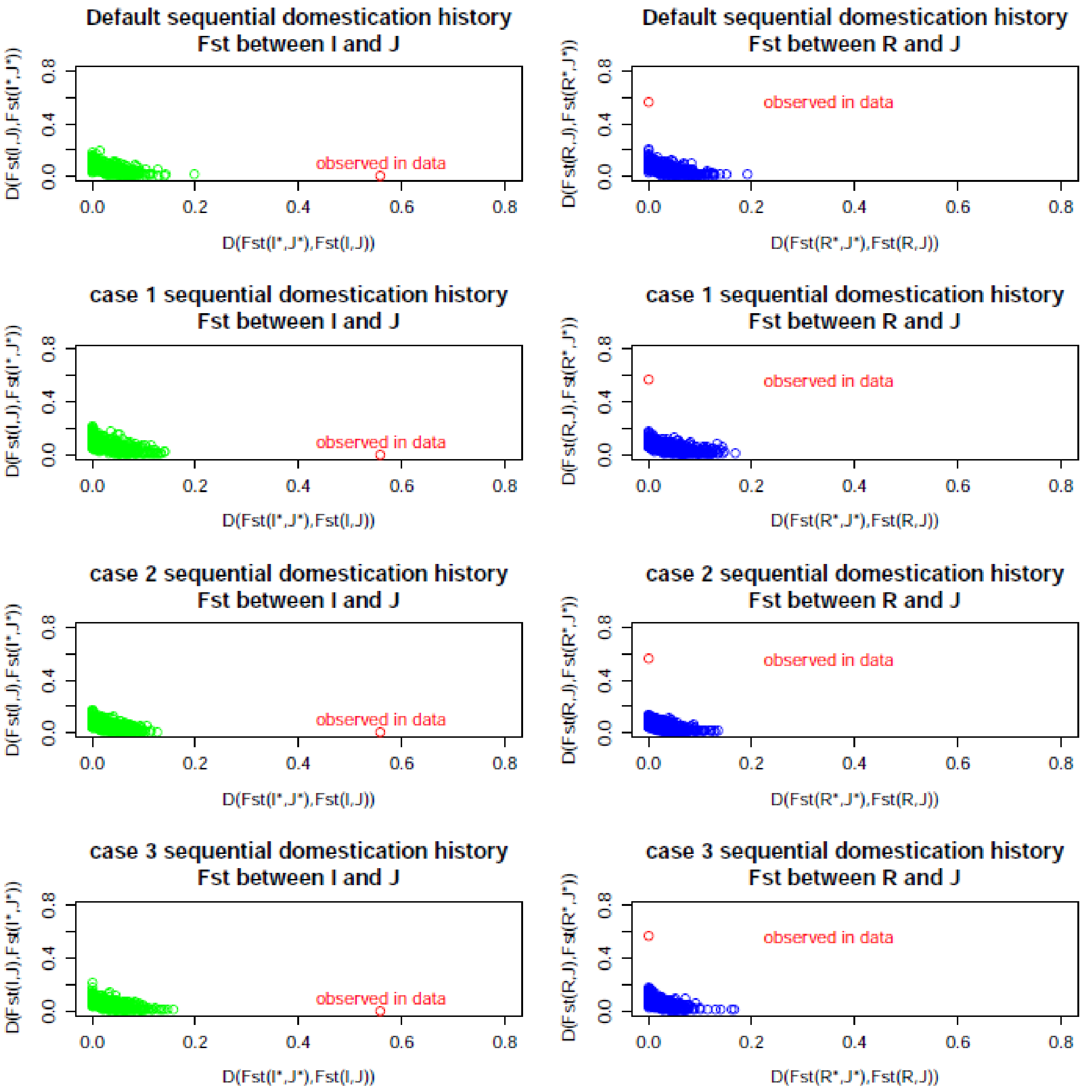

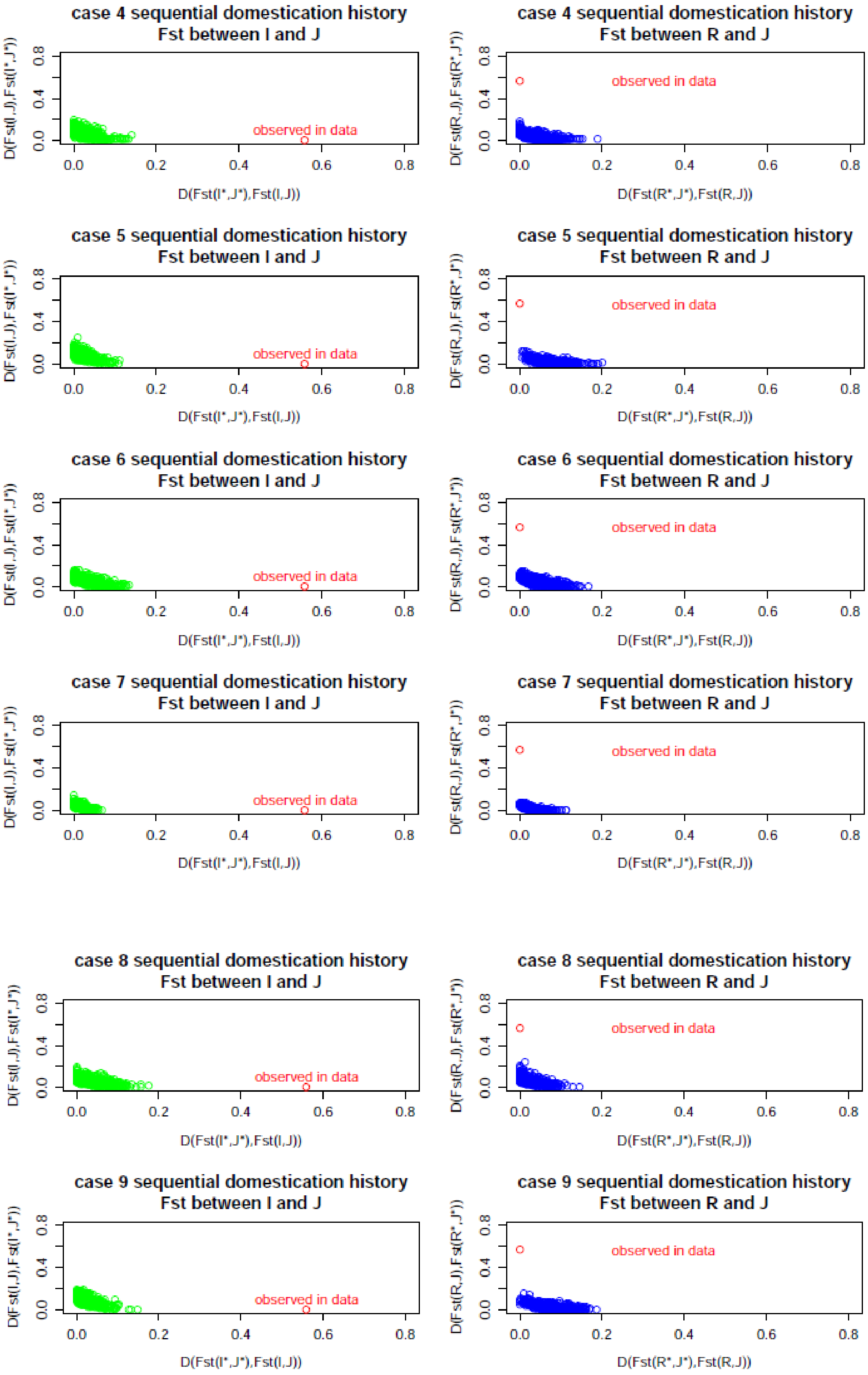
case 4 sequential domestication history
Fst between I and J
observed in data
D(Fst(I*,J*),Fst(I,J))
D(Fst(I,J),Fst(I*,J*))
case 4 sequential domestication history
Fst between R and J
observed in data
D(Fst(R*,J*),Fst(R,J))
D(Fst(R,J),Fst(R*,J*))
case 5 sequential domestication history
Fst between I and J
observed in data
D(Fst(I*,J*),Fst(I,J))
D(Fst(I,J),Fst(I*,J*))
case 5 sequential domestication history
Fst between R and J
observed in data
D(Fst(R*,J*),Fst(R,J))
D(Fst(R,J),Fst(R*,J*))
case 6 sequential domestication history
Fst between I and J
observed in data
D(Fst(I*,J*),Fst(I,J))
D(Fst(I,J),Fst(I*,J*))
case 6 sequential domestication history
Fst between R and J
observed in data
D(Fst(R*,J*),Fst(R,J))
D(Fst(R,J),Fst(R*,J*))
case 7 sequential domestication history
Fst between I and J
observed in data
D(Fst(I*,J*),Fst(I,J))
D(Fst(I,J),Fst(I*,J*))
case 7 sequential domestication history
Fst between R and J
observed in data
D(Fst(R*,J*),Fst(R,J))
D(Fst(R,J),Fst(R*,J*))
case 8 sequential domestication history
Fst between I and J
observed in data
D(Fst(I*,J*),Fst(I,J))
D(Fst(I,J),Fst(I*,J*))
case 8 sequential domestication history
Fst between R and J
observed in data
D(Fst(R*,J*),Fst(R,J))
D(Fst(R,J),Fst(R*,J*))
case 9 sequential domestication history
Fst between I and J
observed in data
D(Fst(I*,J*),Fst(I,J))
D(Fst(I,J),Fst(I*,J*))
case 9 sequential domestication history
Fst between R and J
observed in data
D(Fst(R*,J*),Fst(R,J))
D(Fst(R,J),Fst(R*,J*))
0.0 0.2 0.4 0.6 0.8

Figure S9. Distances in Fst distributions between genomic background and overlapping LDRs for different sequential domestication histories

E. Known genes in overlapping LDRs

Out of the 61 candidate introgression LDRs, we noticed two very interesting genes that have been extensively surveyed and studied world-wide. First, we identified PROG1, a gene associated with the transition from the prostrate growth of ancestral wild rice to the erect growth of cultivars. Interestingly, this gene was mapped using a set of Chromosome Segment Substitution Lines (CSSLs) from a cross between one type of *indica* (Teqing) and wild rice[8]. Sequence comparisons using 87 *indica* and 95 *japonica* cultivars found identical mutations in the PROG1 coding region which suggested common ancestry for this gene[8]. Genetic studies show that selected mutations disrupt the PROG1 gene function and inactivate its expression, leading to erect growth, greater grain number and higher grain yield in cultivated rice[8,9]. As we show in Figure S10 (right panels), a valley of Fst (I,J) around this locus is readily detectable. The sequence patterns around this locus strongly support an evolutionary trajectory where the selected allele first appeared in one domesticated rice species and was subsequently introgressed into all rice cultivars.

The second locus is sh4, a gene that is responsible for seed shattering. One classical trait with cereal crop cultivars is reduction in seed shattering, increasing harvest efficiency during farming. This gene was first broadly located by a QTL study using a cross between *indica* and *rufipogon* and subsequently cloned[10]. Interestingly, the non-shattering allele is present in all 96 *indica* and 112 *japonica* surveyed world-wide[11]. Although the exact function of this gene is not yet known, it is predicted to be a transcription factor involved in cell wall degradation and formation of the abscission layer[10-12]. Similar to what is observed with PROG1, the Fst pattern surrounding this locus also supports an evolutionary history where gene pieces were introgressed from one rice species to the other (chromosome 4 in Figure S1).

In addition to the two genes discussed above, there is another important gene Rc, a bHLH (basic Helix-Loop-Helix) protein that regulates anthocyanin biosynthesis in the seed coat. While wild rice and some *sativa* land-races have red pigment in the pericarps, most of the cultivars are white in the seed. A genetic study showed that 98% of white rice cultivars bear a 14bp deletion that is thought to be the causative mutation for white pericarp. Moreover, sequence patterns show a clear signal of introgression from the *japonica* population to *indica*[13]. Our data in Figure S10 also support this history of introgression as we can see a valley of low Fst(I,J) around this gene. However, this gene is not within the overlapping LDRs we identified. This is because the reduction in diversity in *indica* is not large enough to drive the diversity below the nominal cutoff. Even though the selective sweep is almost complete in many white rice populations, there are still some populations, belonging to the aus subgroup (*indica* subpopulation), that have intermediate frequencies of the selected allele[13]. More importantly, many cultivar populations, including several samples from the current study, are of red pericarp subtypes (Table S1), where the focal selected allele is absent. This is especially true for

some of the *indica* populations (Table S1). The existence of the incomplete sweep in *indica* is the major reason why the diversity in *indica* is insufficiently reduced to be classified as an LDR according to our criteria.

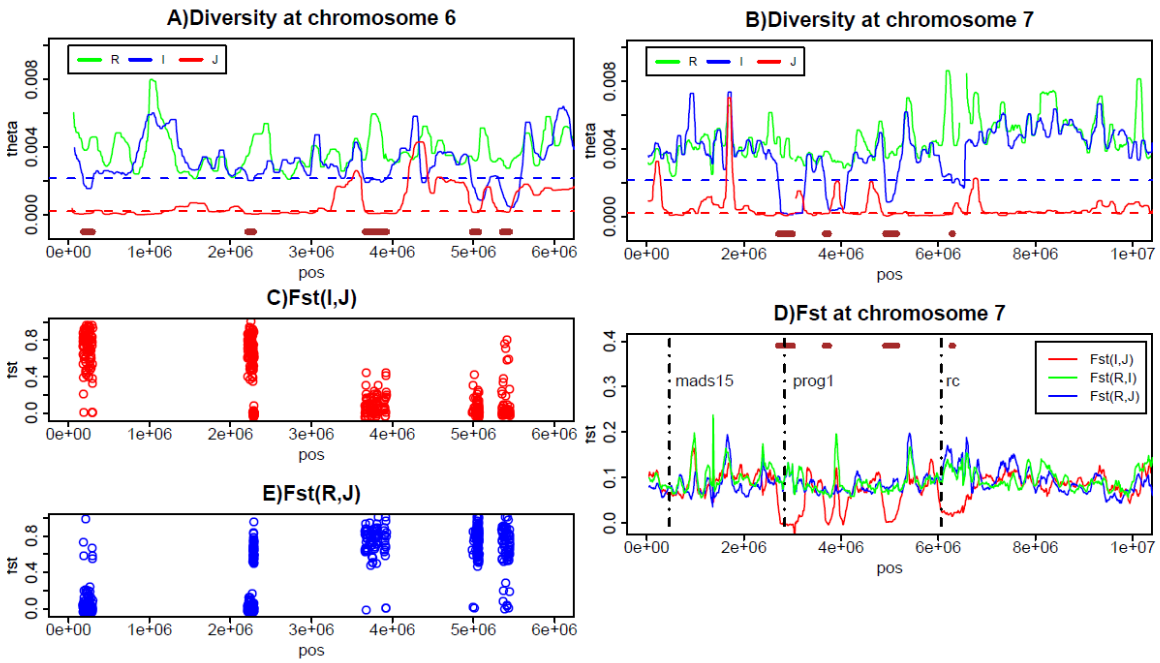

Figure S10. Diversity for three species at chromosome 6 (A) and 7 (B), Fst plot for sites in overlapping LDRs within chromosome 6 (C, E) and sliding window scan for mean Fst values along the chromosome 7 (D). The top panels show the theta estimates (100kb windows) for three populations at chromosome 6 and 7. Genome wide cutoffs are drawn in dashed lines. Overlapping LDRs are shown in horizontal brown bars. The left bottom two panels plot the Fst between I,J and R,J for sites in overlapping LDRs (only Fst(R,I) > 0.5 are shown, see text). The right bottom panel shows the sliding window (100kb window stepping at 10kb) estimates of mean Fst value for three pairwise comparisons. The vertical bars indicate the positions of three known genes. Brown segments display the locations for the overlapping LDRs.

F. The pattern of Fst distributions is robust to cutoffs we used

The cutoffs we used throughout this study are based on shuffling the whole genome and picking the bottom 5% across the replicated genome. As we will show in this section that, if we choose other sets of criteria to identify genome wide low diversity regions, the pattern we saw are qualitatively very similar to Figure 4A and 4B.

For example, if we use 5% or 10% quantile of the genome wide diversity from *indica* and 10% or 20% quantile of the *japonica*, we plot the cumulative distributions of Fst for

genome wide and overlapping LDRs in Figure S11. We observe very similar pattern to Figure 4A and 4B. Thus the observed pattern in Figure 4 is robust to what cutoffs we used to identify LDRs.

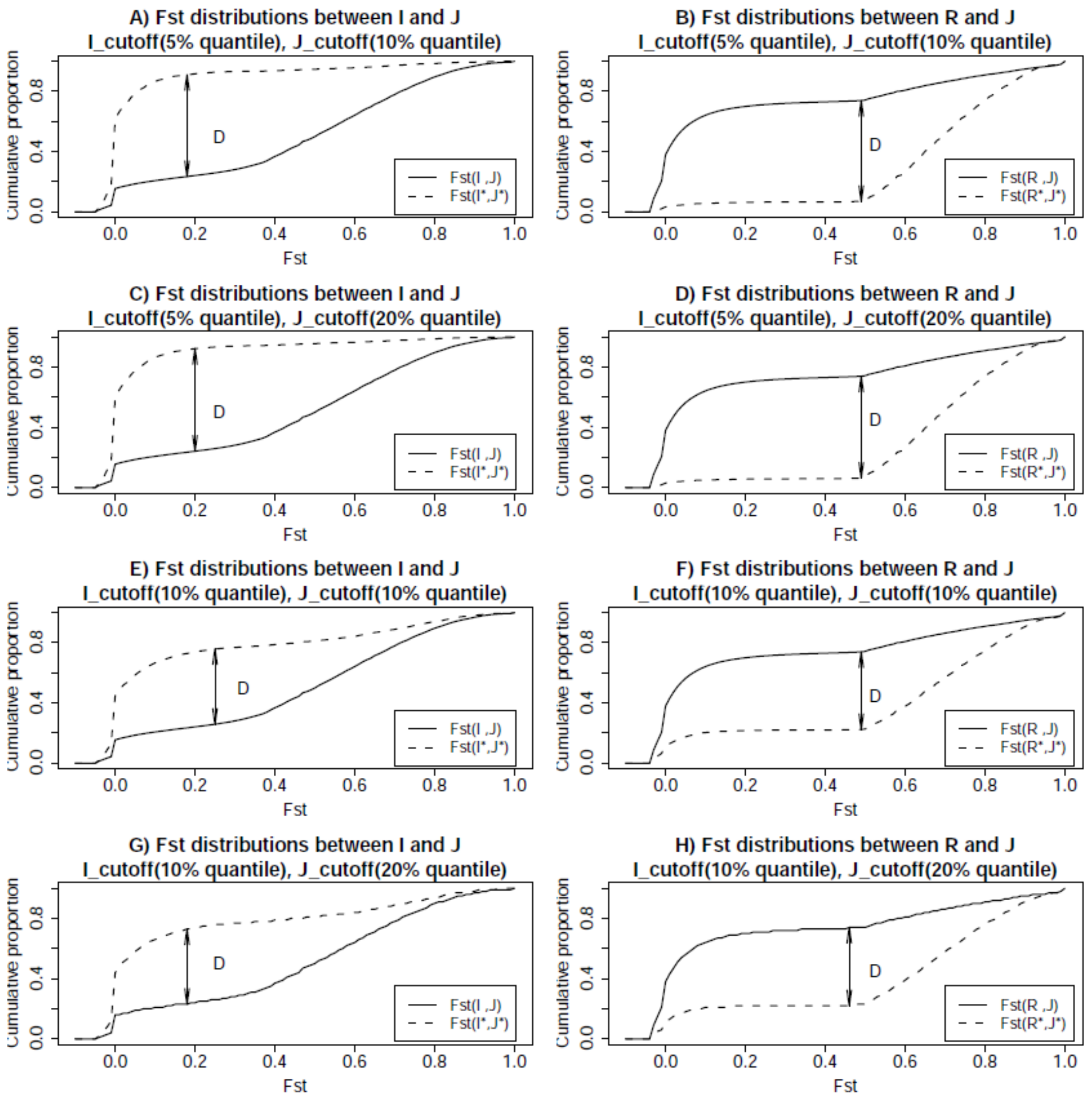

Figure S11. The Fst distributions under different cutoffs for *indica* and japonica. A) and **B)** are using 5% quantile in *indica* and 10% quantile in *japonica*; **C)** and **D)** are using 5% quantile in *indica* and 20% quantile in *japonica*; **E)** and **F)** are using 10% quantile in *indica* and 10% quantile in *japonica*; **G)** and **H)** are using 10% quantile in *indica* and 20% quantile in *japonica*.

G. The conclusions are robust to population structure in *rufipogon*

All of the domestication models used in previous simulations assumes a large panmictic population of O. *rufipogon* from which the two domesticated subspecies are derived. A natural question is that whether population differentiation across the ancestral O. *rufipogon* population that could affect the conclusions of our results. This section will be dedicated to answer this question.

Suppose that the ancestral *rufipogon* population was structured and *indica* was domesticated from a subpopulation R_1' and *japonica* was domesticated from R_2'(Figure S12). The ancestral R1' gave rise to the extant R_1, and likewise R_2' gave rise to R_2.

In this scenario, the distance between *indica* and *rufipogon* is an average between *indica* and R_1 and *indica* and R_2. In other words, d(I, R)= 0.5* d(I, R_1) + 0.5* d(I, R_2). Since d(I, R_1)=2d_2 and d(I, R_1)=2d_1+2d_2, the distance between *indica* and *rufipogon* d(I,R) is then d1+2d2.

The distance between the two cultivars *indica* and *japonica* d(I, J) =2d_1+2d_2 > d(R, I)= d_1+2d_2. In similar fashion, we can also prove that d(I, J) > d(R, J). So, our argument that in the independent domestication history, d(I, J) >=d(R, I) and d(I, J) >=d(R, J) are still true in the presence of population substructure.

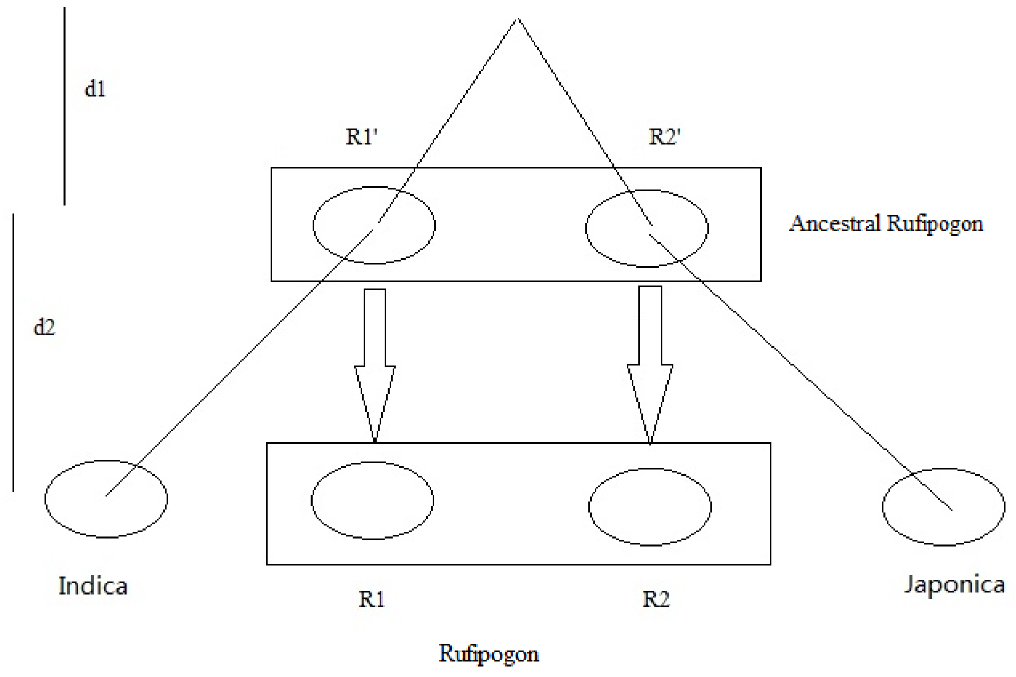

Figure S12. A possible rice demography with population structure in *rufipogon*

In the sequential domestication history, it can also be shown that, the relationship that d(I, J) <= d(R, I) and d(I, J) <= d(R, J) also hold in the presence of population structure.

In summary, if the ancestral O. *rufipogon* population is structured, the P value associated with the rejection of independent domestication model for the overlapping LDRs would be even smaller than reported. Therefore, our conclusions are conservative in the presence of ancestral population structure.

H. Introgression directionality and time

The main point of this study is that certain LDRs appear to be introgressions driven by positive selection. Another interesting, but secondary question is the direction and timing of introgression. Since introgression, like gene migration between populations, means that two geographical or racial samples are the same, the direction of introgression has to be inferred from the genetic background, rather than the introgressions themselves. Our criteria for the inclusion of these secondary points are, hence, that they can be presented succinctly and convincingly. Unfortunately, neither criterion can be met.

In theory, the introgression occurred as two events of selective sweep – the primary selective sweep in one species and then the secondary sweep after introgression. To infer the direction of introgression would entail distinguishing the primary from the secondary sweeps. The ratios should be reciprocal in the two subspecies. For example, if they are in 7:3 ratio in *indica*, they should be roughly 3:7 in *japonica*. We reason that the footprint of the secondary sweep would generally be larger than that of the primary sweep. In the primary event, selection may proceed at the rate comparable with, or slightly faster than, other known sweep events. In contrast, the secondary sweep resulting in introgression may be caused by breeders' decision to select for a known and desired trait and this process should progress very rapidly. The reasoning above would separate the introgressions into 2 bimodal groups. If we rank the introgressions by their sizes in *indica* and also by their sizes in *japonica*, we might detect two clusters. In one cluster, the size ranks would be relatively small in *indica* and relatively large in *japonica*, this is the I-to-J group. The other cluster, the J-to-I group, would show the opposite trend.

When we carried out this analysis, the distinction is rather weak. There are many population genetic reasons for the lack of statistical power in resolving the two clusters. One of them is that *japonica* is generally much lower in polymorphism than *indica* or *rufipogon* genome-wide. The statistical resolution is too weak to be conclusive here in our study.

Supporting References

1. Ma J, Bennetzen JL (2004) Rapid recent growth and divergence of rice nuclear genomes. Proc Natl Acad Sci U S A 101: 12404-12410.

2. Vitte C, Ishii T, Lamy F, Brar D, Panaud O (2004) Genomic paleontology provides evidence for two distinct origins of Asian rice (Oryza sativa L.). Mol Genet Genomics 272: 504-511.

3. Zhu Q, Ge S (2005) Phylogenetic relationships among A-genome species of the genus Oryza revealed by intron sequences of four nuclear genes. New Phytol 167: 249-265.

4. Caicedo AL, Williamson SH, Hernandez RD, Boyko A, Fledel-Alon A, et al. (2007) Genome-wide patterns of nucleotide polymorphism in domesticated rice. PLoS Genet 3: 1745-1756.

5. Cao ZH, Ding JL, Hu ZY, Knicker H, Kogel-Knabner I, et al. (2006) Ancient paddy soils from the Neolithic age in China's Yangtze River Delta. Naturwissenschaften 93: 232-236.

6. Hudson RR (2002) Generating samples under a Wright-Fisher neutral model of genetic variation. Bioinformatics 18: 337-338.

7. Mather KA, Caicedo AL, Polato NR, Olsen KM, McCouch S, et al. (2007) The extent of linkage disequilibrium in rice (Oryza sativa L.). Genetics 177: 2223-2232.

8. Tan L, Li X, Liu F, Sun X, Li C, et al. (2008) Control of a key transition from prostrate to erect growth in rice domestication. Nat Genet 40: 1360-1364.

9. Jin J, Huang W, Gao JP, Yang J, Shi M, et al. (2008) Genetic control of rice plant architecture under domestication. Nat Genet 40: 1365-1369.

10. Li C, Zhou A, Sang T (2006) Rice domestication by reducing shattering. Science 311: 1936-1939.

11. Lin Z, Griffith ME, Li X, Zhu Z, Tan L, et al. (2007) Origin of seed shattering in rice (Oryza sativa L.). Planta 226: 11-20.

12. Onishi K, Takagi K, Kontani M, Tanaka T, Sano Y (2007) Different patterns of genealogical relationships found in the two major QTLs causing reduction of seed shattering during rice domestication. Genome 50: 757-766.

13. Sweeney MT, Thomson MJ, Cho YG, Park YJ, Williamson SH, et al. (2007) Global dissemination of a single mutation conferring white pericarp in rice. PLoS Genet 3: e133.
